# Variation of gene expression in plants is influenced by gene architecture and structural properties of promoters

**DOI:** 10.1371/journal.pone.0212678

**Published:** 2019-03-25

**Authors:** Sanjukta Das, Manju Bansal

**Affiliations:** Molecular Biophysics Unit, Indian Institute of Science, Bangalore, Karnataka, India; National Institute of Plant Genome Research, INDIA

## Abstract

In higher eukaryotes, gene architecture and structural properties of promoters have emerged as significant factors influencing variation in number of transcripts (expression level) and specificity of gene expression in a tissue (expression breadth), which eventually shape the phenotype. In this study, transcriptome data of different tissue types at various developmental stages of *A*. *thaliana*, *O*. *sativa*, *S*. *bicolor* and *Z*. *mays* have been used to understand the relationship between properties of gene components and its expression. Our findings indicate that in plants, among all gene architecture and structural properties of promoters, compactness of genes in terms of intron content is significantly linked to gene expression level and breadth, whereas in human an exactly opposite scenario is seen. In plants, for the first time we have carried out a quantitative estimation of effect of a particular trait on expression level and breadth, by using multiple regression analysis and it confirms that intron content of primary transcript (as %) is a powerful determinant of expression breadth. Similarly, further regression analysis revealed that among structural properties of the promoters, stability is negatively linked to expression breadth, while DNase1 sensitivity strongly governs gene expression breadth in monocots and gene expression level in dicots. In addition, promoter regions of tissue specific genes are found to be enriched with TATA box and Y-patch motifs. Finally, multi copy orthologous genes in plants are found to be longer, highly regulated and tissue specific.

## Introduction

Phenotypic alteration depends on gene expression and eventually maintains the complexity of multicellular organism. Variation of gene expression can be measured basically in two ways: one is the concentration of gene product or number of mRNA (messenger RNA) which is considered as the level of expression and other is the number of tissues in which a gene is expressed, which is known as the breadth of expression. However, both the level and breadth of gene expression outline the diversity in the functions and development of specific tissues [1, 2]. In human, chicken and in other metazoans compactness of genes, gene length, coding DNA sequence (CDS) length and all parameters related to the size of intron are found to be negatively related to expression level and expression breadth [3]. However, in case of plants such as arabidopsis and rice, highly expressed genes are observed to have longer intron, CDS and untranslated region (UTR) compared to the lowly expressed ones [4]. Although, in a combined study of expression level and breadth, these above parameters are positively correlated to expression breadth [5, 6]. Furthermore, a study on arabidopsis showed that highly expressed pollen genes have small intron size compared to genes in sporophytes, which essentially points to a different selection mechanism for gametophytes and supports the model of selection for economy [7]. Similarly, studies on a higher plant like soybean showed that lowly expressed genes are longer in length compared to highly expressed genes which also supports the selection for economy model [8].

In addition to the above factors, in higher organisms, GC content of genome varies considerably between the genic and intergenic region. GC-rich regions are also the likely regions of high recombination which enforce mutational biases on the highly expressed genes [9, 10]. Direct correlation of gene expression breadth and GC composition was reported by Lercher et al. in humans and it was supported by *in vivo* experiment on high expression of GC-rich genes [11, 12]. Moreover, similar results were also reported by Rao et al in chicken which demonstrate that 10% of the variation in gene expression is influenced by GC content of genes [13]. However, in plants, while the relationship between G+C content and compactness of gene structure is well studied, the relationship between GC content and gene expression is not yet explored, particularly in higher plants, except for study on arabidopsis [5, 14]. The observed correlation between gene expression measures and gene architecture in terms of length of coding and non-coding region show opposite trends in plants and animals. The present work addresses this issue in higher plants by simultaneous analysis of length and GC content of gene components and their variation with the two gene expression measures, expression level and breadth.

Apart from the G+C composition of gene sequence, promoter sequences and their structural properties have also been linked to expression parameters by several groups [15–19]. Structural properties of promoters regions of genes with low variability of expression are unique and significantly different from genes showing highly variable expression [17]. Further studies have depicted high correlation between the expression breadth and level in plants and metazoans [6, 16]. In plants, as most of the genomic parameters are linked to both expression level and breadth as well as breath and level are highly correlated thus, it is important to see which of the expression parameters are influenced more strongly than the other when they are studied together. We have used a statistical approach, multivariate multiple regression model to enumerate the relative effect of expression level or breadth for common genic properties.

The presence of *cis*-motifs in promoter regions of gene provide a platform for binding of transcription factors which in general can modulate the gene expression to a large extent. *Cis*-motifs are well understood regulatory motifs, enriched in promoter regions. However, in plants, most of the studies were focused on general motif enrichment in the promoters of expressed gene but a clear picture of specificity of motif for variably expressed genes is missing. Part of this study is focused on identifying the common consensus motif enriched in the promoter sequence of genes that are expressed tissue specifically or broadly in plants. The knowledge of consensus sequence can measure the activity of each promoter, thus the regulation and activity level of individual gene can be fine-tuned by genetic engineering.

Despite the progress in uncovering the relationship of gene expression divergence with gene architecture as well as promoter properties, gene copy number has also been analysed in plants [20–22]. High frequency of whole-genome duplication and single-gene duplication is also frequently observed in plants.

Picea is one of the plants, belonging to the gymnosperm clade which has been studied extensively for the significance of gene copy number in gene expression, rates of sequence divergence, protein length and codon bias. Similarly, individual studies on arabidopsis and rice indicate that duplicate genes or multi copy number genes show divergence in expression rather than singleton (single copy) genes [23–25]. Gene duplication is a principal mechanism for increasing functional diversification in different tissues, however, there are no such comparative studies on gymnosperm which can throw light on the variation of expression with increase in copy number. In gymnosperms, how the genomic parameters shape the evolution of duplicate genes by regulating the expression of genes also need to be explored. In this study, we have tried to address these lacunae by carrying out a comparative analysis of various genomic traits for arabidopsis, rice, sorghum and maize.

## Materials and methods

The genomic DNA sequences, coordinates of primary transcript, UTR, exon, intron and transcription start site (TSS) of *A*. *thaliana* (arabidopsis), *O*. *sativa* (rice), *S*. *bicolor* (sorghum) and *Z*. *mays* (maize) were downloaded from the TAIR10 version 13 (The recent release of TAIR [26], ARAPORT11 [27] has only added missed annotation of noncoding genes), RAPDB server Build 5 (http://rapdb.dna.affrc.go.jp), SbGDB version 2 (version:JGISbi/SbGDB181) and B73 RefGen_v3 (https://www.maizegdb.org/) respectively. Since, mapping file of database KEGG [28] and the molecular and functional pathways are based on the above version, in case of rice, sorghum and maize, the previous version is used rather than the latest one. We used Perl script to extract sequences from its respective coordinates and for the calculation of GC percentage as well as length.

### Microarray data analysis

Gene expression information for arabidopsis, rice, sorghum and maize were downloaded from AtGenExpress [29], Gene Expression Omnibus [30], Gene Expression Omnibus [31] and MaizeGDB [32] respectively for developmental stages. Treated tissue samples were removed from this analysis, thus, only 63 tissue samples in arabidopsis, 29 tissue samples in rice, 15 tissue samples in sorghum and 60 tissue samples in maize were included for further analysis. Signal intensities captured from the same tissue were averaged. Genes are considered as expressed in tissues if the signal intensity values are above 200. The distributions of genes before and after using cut-off are shown in S1 Fig. Gene expression level is calculated for a gene by averaging the intensity values in all tissues. Gene expression breadth is measured as the number of tissues in which a gene is expressed. Thus, genes included in this analysis by using above the cut-off for arabidopsis, rice, sorghum and maize are 15667, 18762, 20461 and 18694 respectively (S1 Fig).

The mean values of expression levels were arranged in ascending order and the cumulative values of different parameters from the top and bottom ends, corresponding to 1%, 5%, 10%, 15%, 20%, 25%, 30%, 35%, 40%, 45% and 50% of the data were calculated, in order to compare the difference between lowly and highly expressed genes. To check the change in different parameters with an increase in average expression value of level and breadth, the expression values were also sorted in an ascending order, then the whole set of data was divided into 10 groups, each containing 10% of the data and the averaged value calculated for different parameters.

### Structural parameters

Four structural parameters were used to analyse the promoter properties such as average free energy (AFE), DNase1 sensitivity, nucleosome positioning preference (NPP) and curvature. The promoter region (-200 to 0 from the TSS) were extracted by using Perl program from the available coordinates of the TSS. Structural parameters are calculated for 1000 nt long genome sequence flanking the TSS (-500 to +500). The details of structural properties analysed are outlined below.

#### Average free energy (AFE)

Stability of double stranded DNA can be measured by its free energy values. It is calculated by summing up the free energies of the constituent base paired dinucleotides. Among total possible 16 dinucleotide steps, 10 are the unique dinucleotide steps. The free energy values for the corresponding 10 dinucleotides were taken from the unified parameters procured from the melting studies on 108 oligonucleotides [33]. In the present analysis average free energy calculated over a 15 base pairs window (or 14 dinucleotide steps) was used for each promoter sequence by assigning the value to the central base pair. For each window, the free energy is calculated with one nucleotide (nt) sliding window for the whole stretch of a promoter sequence.

#### DNase1 sensitivity and Nucleosome position preference (NPP)

Bendability can be induced by the presence of protein in the promoter region. DNase I sensitivity and NPP are trinucleotide based models which have been used for bendability calculations. DNase 1 sensitivity model used the bending propensities of 32 trinucleotide parameters which have been retrieved from a 709 DNase I cutting frequencies study [34], while NPP model measures the trinucleotide bendability parameters for DNA sequences based on nucleosomal positioning preferences [35]. In short, for a given sequence the bendability profiles are calculated by adding up the values of trinucleotide parameters corresponding to each consecutive overlapping trinucleotide with a window size of 30 nucleotides (nt) long sequences and then assigning the value to the middle base pair.

#### Curvature

Curvature is a dinucleotide based parameter that measures the intrinsic sequence dependent bending of DNA in the absence of protein. Dinucleotide wedge angles derived from experimental data on gel retardation assays to train the dataset were used to calculate the bending property of DNA duplex sequences (BMHT parameter) [36]. Here we used in house software NUCRADGEN [37, 38] to calculate the curvature of DNA sequences by using BMHT parameter. Curvature of a DNA fragment is defined by the d/lmax ratio where d is the linear distance between the first and last base pair of the DNA and lmax is the contour length. An overlapping window of 75 nucleotides was used for the calculation of d/lmax. The ratio varies between 0 to 1, where 0 corresponds to closed circle and 1 to the straight DNA molecule.

### Multivariate multiple regression model

Simple linear regression analysis is a statistical approach for enumerating the relationship between dependent and independent variables. Linear regression model is in a compact vector form as

Y=Xβ+ε

Where ‘Y’ is observations of dependent variables in terms of response vector, ‘X’ is representation of independent variables as regressors or predictors, ‘β’ is regression coefficient and ‘*ε*’ as random errors. The multivariate multiple regression model has been built by further including more than one dependent and independent variables as

$$Y_{n\times d}=\;X_{\;n\times \left(p+1\right)}\beta_{\left(p+1\right)\times d}+\varepsilon_{n\times d}$$

Where Y has n × d variables, where n is the number of observations cohere to the number of genes (genes incorporated for arabidopsis, rice, sorghum and maize are 13392, 15310, 17021 and 15295 respectively) and d is the dimensional response. We have used here two dimensional response (d = 2) gene expression level and expression breadth. Change in response vector can be explained by the predictor p (X), in present analysis we included all genomic and promoter parameters as independent variables or predictor variables. In arabidopsis, rice and maize 13 predictors are included (p = 13), while in sorghum p = 11. The matrix is in the form as

$$\left[\begin{array}{c} \begin{array}{cc} y_{11} & y_{12}\\ y_{21} & y_{22}\\ \vdots & \vdots \end{array}\\ \begin{array}{cc} y_{n1} & y_{n2} \end{array} \end{array}\right]=\left[\begin{array}{c} \begin{array}{cc} 1 & x_{11}\\ 1 & x_{21}\\ \vdots & \vdots \end{array}\;\;\;\;\begin{array}{c} x_{12}\\ x_{22}\\ \vdots \end{array}\;\;\;\begin{array}{cc} \ldots & x_{1p}\\ \ldots & x_{2p}\\ \vdots & \vdots \end{array}\\ \begin{array}{cc} 1 & x_{n1} \end{array}\;\;\;\begin{array}{ccc} x_{n2} & \ldots & x_{np} \end{array} \end{array}\right]\;\left[\begin{array}{c} \begin{array}{cc} \beta_{01} & y_{02}\\ \beta_{11} & \beta_{12}\\ \vdots & \vdots \end{array}\\ \begin{array}{cc} \beta_{p1} & \beta_{p2} \end{array} \end{array}\right]+\left[\begin{array}{c} \begin{array}{cc} \varepsilon_{11} & \varepsilon_{12}\\ \varepsilon_{21} & \varepsilon_{22}\\ \vdots & \vdots \end{array}\\ \begin{array}{cc} \varepsilon_{n1} & \varepsilon_{n2} \end{array} \end{array}\right]$$

#### Data normalization

Length associated genomic parameters, such as length of primary transcript (PT), exon and intron as well as gene expression level were log-transformed to improve the interpretability. Moreover, we also analysed the z-values for all predictor variables (independent variables like genomic and promoter parameters) and response variables (dependent variable such as gene expression level and breadth). The z-value for a variable parameter ‘x’ is defined as

$$z=\frac{\left(\text{x}-\mu \right)}{\sigma }$$

Where µ is the mean and σ corresponds to standard deviation. This standardization of data places the mean at 0 and standard deviation as 1 thus facilitating comparison of data.

#### Variance inflation factor (VIF)

VIF is collinearity analytic, calculated to check the multicollinearity among the independent variables or predictors [39–41].

$$VIF_{i}=\;\frac{1}{1-R_{i}^{2}}$$

Variation of predictor i is mainly described by a linear combination of the other predictors, when the R_i_^2^ is close to 1, the VIF for that predictor increases correspondingly, thus, VIF value becomes 1 when the correlation among predictors is zero. In the present study, a stringent cut off of VIF ≤ 5 is considered further for predictors in building the model since regression coefficient (β) value becomes high due to of high correlation among predictors (multicollinearity), which makes the results erroneous. The VIF values are listed before and after removing the predictors in the S1 Table.

The regression coefficient (β), the slope of the regression line is a quantitative measure between response variables and predictors. Moreover, coefficient estimates the change in response variable per one-unit change in one predictor while other predictors are held constant and the sign of β implies the direction of X and Y (with a positive coefficient implying X and Y change in the same direction and vice versa). However, estimating the results of multiple regression models based on its value can be biased, so *p*-values were considered further to rigorously evaluate the results. Since multiple hypotheses are compared here, we have used Bonferroni correction [42] to test the significance level of the *p*-values which minimize the type-1 error (incorrect rejection of false positive).

### Enrichment of hexamer motif

Enriched hexamer motifs were screened in order to trace possible binding sites for transcription factors in the promoter regions (-200 upstream to 0 with respect to TSS) of broadly and narrowly expressed datasets. Frequency of occurrence of all possible hexamers was calculated, using a 1 nucleotide sliding window for both the datasets. The hexamer frequency of broadly expressed genes (top 10%) was then compared with the frequency of narrowly expressed (bottom 10%) dataset. Hexamer frequency calculation was done using a Perl script. MATLAB R2011b was used to plot hexamer frequencies and to identify hexamers whose frequency sequence differed by >2σ from the best fit line. PLACE database was used to screen consensus motifs of transcription factors (http://www.dna.affrc.go.jp/PLACE/) for a match between the *cis*-binding elements and the enriched hexamer sequences derived from the analysis.

### Identification of orthologous group or gene family

In the present analysis, PLAZA 3.0 [43] database was used to analyse the comparative genomics between arabidopsis, rice, sorghum and maize. Orthology information was downloaded from the integrative orthology data of PLAZA. This database uses OrthoMCL [44] to cluster an orthologous group (gene family), which uses BLASTP [45] with an all-against-all option and an e value cut-off of 1e−05; therefore Orthologs detected through OrthoMCL clustering were used further in our analysis. Grouping was done based on the number of genes present in orthologous group. Rice was used as the reference genome to recognize one-to-one orthologs of single-copy gene (singleton gene), two-copy genes, 3–5 genes and >5 genes in arabidopsis, sorghum and maize, whereas for rice orthologous grouping was based on orthologs common to all plants (S2 Table).

### Enrichment of functional categories

Expression data were sorted in an increasing order of number of tissue samples and top and bottom 25% of the data were classified as narrowly expressed and broadly expressed respectively. As information of GO terms is available only for limited number of genes, we have used here 25% of the data while 10% of the data was used for rest of analysis. Functional categories of the orthologous group were also analysed in rice. PageMan application of MapMan was used to evaluate statistically significant GO terms as per the hypergeometric test [46]. Calculation of *p*-value from the Z-score value and generation of color map was performed using MATLAB. GO terms with *p*-value 0.05 were considered as significant.

## Results and discussion

Comparative analysis of the four plant genomes illustrates that rice genome is 4 times larger than arabidopsis, while being 3 and 10 times smaller than sorghum and maize respectively. Interestingly, length of coding region (total exon coverage as percentage of primary transcript) becomes smaller with an increase in genome size whereas the intron size increases with genome size and hence the median length of the primary transcript (PT) does not show much difference across these plants (Table 1). A distinct difference was seen in the GC content of UTR regions and in the intergenic region between dicot and monocot. The GC% difference between 5’ UTR and intergenic region is 6% in arabidopsis while in monocots it is >10%. Within the genic region, GC-content of 5’ UTR is more than that of the coding region (exon) in monocots whereas an opposite trend is observed in arabidopsis (Table 1) as reported in an earlier study [47].

**Table 1:** Comparative statistics of various parameters of whole genome and gene architecture of arabidopsis, rice, sorghum and maize.

	Arabidopsis (119 Mb)	Rice (382 Mb)	Sorghum (730 Mb)	Maize (2400 Mb)
Gene density (genes per MB)	238.8 app.	101.45 app.	49.1 app.	16.3 app.
Transcribed region	40.00%	30.40%	14.46%	6.70%
Exon coverage	20.30%	15.34%	6.60%	2.44%
Protein coding genes^a^	27169	37869	35849	39149
Average GC content	36.00%	43.56%	43.8%	46.9%
Median length of various region in protein coding genes^a^ (% of primary transcript length)				
primary transcript	2095	2317	2152	2511
Intergenic	924	8560	10803	56138
5' UTR	105 (6.1%)	97(7.2%)	102(1.9%)	163(4.2%)
3' UTR	208 (9.5%)	227 (13.6%)	271(4.5%)	305(6.7%)
Intron	705(32.2%)	1501(46.5%)	1364(54.1%)	1593(57.4%)
Exon	1332(66.8%)	1358(50.6%)	1218(45.9%)	1351(36.3%)
Average GC % of various regions in protein coding genes^a^				
primary transcript	39.2 ± 3.0	49.16 ± 10.18	46.5 ± 10.4	48.2 ± 9.3
Intergenic	31.9 ± 4.9	42.27 ± 4.7	42.6 ± 4.8	46.2 ± 4.1
5' UTR	37.7 ± 8.0	54.77 ± 18.2	60.4 ± 11.8	57.2 ± 11.6
3' UTR	31.7 ± 4.8	42.51 ± 9.36	40.8 ± 6.8	41.7 ± 9.7
Intron	32.6 ± 4.2	42.67 ± 11.73	38.7 ± 7.5	39.7 ± 7.2
Exon	42.5 ± 3.0	52.2 ± 8.8	52.7 ± 9.5	52.9 ± 8.5

^a^ corresponds to the annotated TSS data set. Concatenated length and GC% values are tabulated for all coding and noncoding region of the genes.

### Gene components variation with expression level and breadth

**Fig 1:**
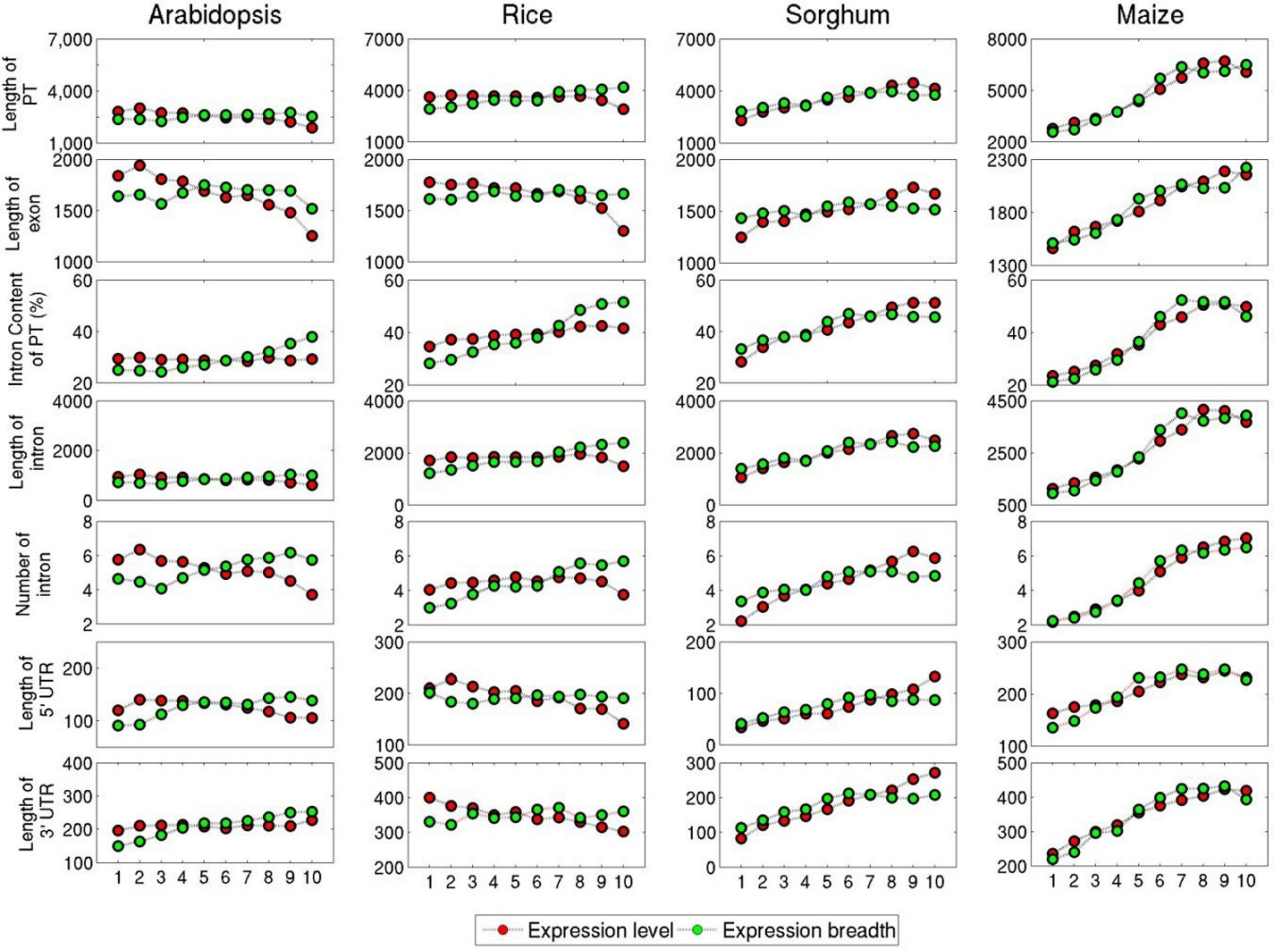
The relationship between 7 different length parameters of plant genes and their expression values. Each panel shows bin number (each containing 10% data) on x-axis versus the average parameter values with increase in expression level and breadth on y-axis. The dataset is divided into 10 equal bins with 10% of data in each bin. Red and green color dots represent expression level and expression breadth respectively.

**Fig 2:**
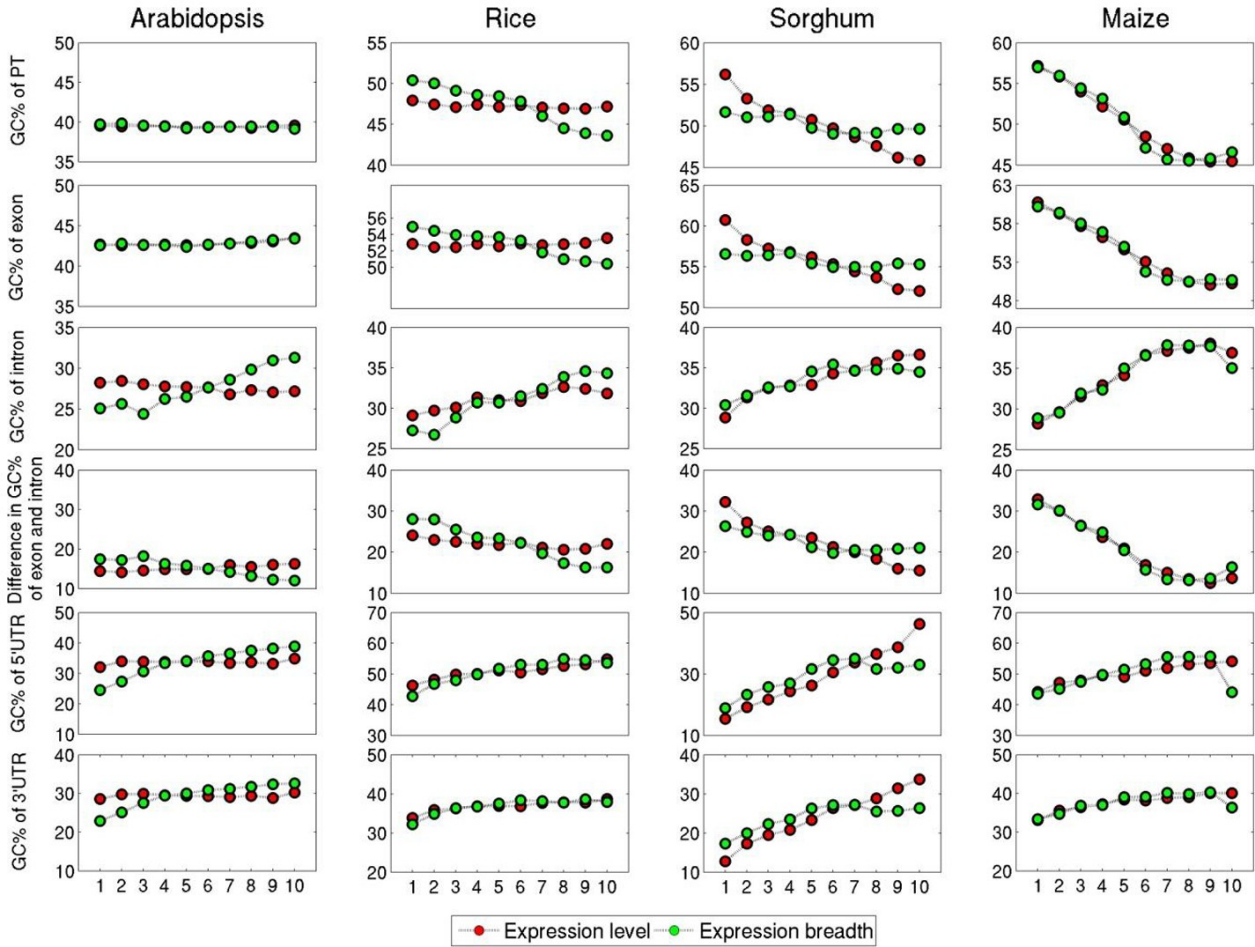
The relationship between 6 different G+C content parameters of plant genes and their expression values. Each panel shows a bin number on x-axis versus the average parameter values with increase in expression level and breadth on y-axis. The dataset is divided into 10 equal bins with 10% of data in each bin. Red and green color dots represent expression level and expression breadth respectively.

In this study, we have analysed two parameters related to gene components with respect to the average expression level and breadth. First is the length parameter which includes length of primary transcript, length of concatenated exon, intron content of primary transcript (%), length of concatenated intron, number of introns, lengths of 5’ UTR and 3’ UTR. Second parameter is associated with GC content of coding and noncoding regions, such as GC% of primary transcript, GC% of the concatenated intron, GC% of the concatenated exons and difference in GC% of concatenated exon and intron and GC% of 5’ UTR and 3’ UTR. We have analysed these parameters for their correlation with increase in gene expression level and breadth by dividing into 10 bins, each with 10% percentile data (Figs 1 and 2). The Pearson’s correlation coefficient was calculated between each parameter and the expression values and is listed in Table 2. This study revealed that length of primary transcript, intron content of primary transcript, length of intron, number of introns and length of 5’ UTR and 3’ UTR are significantly positively correlated (*p* < 0.0001) with the expression breadth in all plants. However, the correlation value of length of exon and expression breadth is trivial in arabidopsis (0.003) and in sorghum (0.016) (Table 2). The expression level and expression breadth showed a parallel trend for all length related parameters for sorghum and maize (Fig 1) while in arabidopsis and rice correlation is noticed to be opposite for the same parameters, excluding length of 5’ UTR and 3’ UTR (Table 2). Similar results were reported earlier in arabidopsis and rice [5, 6]. Our results have highlighted that in monocots, expression breadth showed a similar profile (positive correlation) with respect to length of coding and noncoding region, while profiles of expression level are similar for arabidopsis and rice. Earlier studies on human and arabidopsis have reported that intron density is positively linked to expression level and breadth [5, 6, 48, 49]. The positive correlation of intron density and gene expression in human and arabidopsis implies a biasness towards intron gain for broadly expressed genes and some studies have indeed suggested a gain in introns during evolution in highly expressed genes [50].

**Table 2:** Pearson’s correlation coefficient values for gene expression measures vs gene architecture as well as promoter properties are presented here.

	Arabidopsis		Rice		Sorghum		Maize	
	Level	Breadth	Level	Breadth	Level	Breadth	Level	Breadth
Length of PT	0.107	-0.207	0.211	-0.071	0.107	0.215	0.442	0.389
Exon length	0.003*	-0.228	0.025	-0.168	0.016*	0.118	0.262	0.248
Intron con.	0.200	-0.003*	0.262	0.071	0.147	0.164	0.359	0.266
Intron length	0.152	-0.143	0.233	-0.028	0.147	0.200	0.431	0.337
Intron no.	0.070	-0.143	0.133	-0.052	0.085	0.166	0.277	0.293
5’ UTR length	0.281	0.035	0.116	0.051	0.150	0.301	0.201	0.172
3’UTR length	0.316	0.069	0.149	0.063	0.162	0.302	0.272	0.248
GC% of PT	-0.043	0.013*	-0.276	-0.021*	-0.074	-0.267	-0.499	-0.475
Exon GC%	0.100	0.109	-0.204	0.037	-0.056	-0.259	-0.483	-0.476
†Intron GC%	**0.247**	**-0.113**	-0.023*	**0.048**	**0.034**	**-0.109**	**-0.208**	**-0.229**
Intron GC%	**0.195**	**-0.032**	**0.165**	**0.055**	**0.095**	**0.125**	**0.181**	**0.175**
Diff. In GC%	-0.145	0.191	-0.146	0.045	-0.112	-0.129	-0.278	-0.227
5’ UTR GC%	0.312	0.030	0.154	0.097	0.157	0.288	0.161	0.138
3’UTR GC%	0.292	0.022*	0.130	0.094	0.151	0.282	0.136	0.126
AFE	-0.058	-0.021*	-0.083	-0.111	-0.069	-0.010*	0.098	0.094
Dnase 1	-0.101	0.089	-0.159	0.027	-0.124	-0.106	-0.231	-0.164
NPP	0.114	-0.034	0.072	0.008*	0.083	0.065	0.137	0.105
Curvature	-0.012*	0.035	-0.005*	0.054	0.017*	-0.025	-0.124	-0.095

Insignificant values at p<0.001 are marked with *.

†Intron GC% is for dataset which includes single exon genes. The correlation coefficient values of GC% of intron (datasets including and excluding single exon genes) with expression level and breadth are presented in bold.

In addition to the length parameters, an earlier study on a few human genes had revealed that GC content of a primary transcript plays a critical role in its expression [11] while a direct correlation was also seen in avian genes [13]. Furthermore, in green algae it is reported that GC content and codon usage helps in chromatin modification, thus regulating gene expression [51], while in grass family the GC% of genes is heterogeneous and high GC content genes show more variable expression [14]. Hence further analysis was carried out to check the influence of GC% of primary transcript and its components on expression of genes in plants. Results illustrated that in monocots, gene expression (level and breadth) parameters are negatively linked with GC% of primary transcript, GC% of concatenated exon and difference in GC% of concatenated exon and intron, whereas, they are positively correlated with GC% of intron for the full set of genes (including all zero intron genes) (Fig 2). Possibly, as a consequence of this, the GC% of primary transcript is significantly correlated with GC% of exon. Surprisingly in grasses, if zero intron (single exon) genes were removed from the analysis, GC% of intron in remaining genes showed a negative correlation with one or both of the gene expression parameters, such as level in rice, breadth in sorghum and both level and breadth in maize (Table 2). However, in arabidopsis no such difference was noticed before and after removal of zero intron genes. In arabidopsis, similar relationship between GC% of intron and gene expression has been reported earlier [5]. Zero intron genes were hence excluded in further analysis from parameters related to intron.

In all studied plant systems, GC% of 3’ UTR and 5’ UTR showed significant positive relationship with expression level and breadth (p < 0.0001) although in arabidopsis, the relationship between expression level and 3’ UTR is not significant (Table 2). Summarising the above results on grass family, GC% of primary transcript, GC% of coding and noncoding regions are negatively linked to gene expression parameters while 5’ UTR is positively correlated to gene expression parameters. Similar results were also reported for avian genome [13] which also has heterogeneous genomes in terms of GC composition of genes [52]. The correlation of expression breadth was found to be stronger than that for expression level for all gene components as seen in Fig 2, where both are plotted together (Fig 2). The absolute differences in gene components were smaller for gene expression level as compared to gene expression breadth in arabidopsis and rice and highly expressed genes are more compact. It may be mentioned that an earlier study of arabidopsis and rice had reported opposite results viz. highly expressed genes are longer compared to lowly expressed genes [4]. Therefore, quantile analysis was performed on gene expression level by taking the sorted data in ascending order and 1%, 5%, 10%, 15%, 20%, 25%, 30%, 35%, 40%, 45% and 50% quantiles for both sides of the whole data set were compared (S2 and S3 Figs). No significant difference was apparent from Figs 1 and 2 but a clear separation in values of gene components between highly and lowly expressed genes was seen in S2 and S3 Figs. The differences between various parameter values for high and low expression level in arabidopsis, rice, sorghum and maize were found to be significant at *p* value ≤ 0.0001 (Kolmogorov-Smirnov (KS) test was performed at 25% quantile level). Mean value at 25% quantile level for all four plants is shown in Table 3. Compiling the above results on relationship between variation of expression parameters with respect to length and GC% of gene components, we concluded that significant amount of variation in gene expression level and breadth can be predicted from their genomic parameters and a plethora of relationships are present between the genomic parameters and the expression parameters in plants. Among all gene components studied here, the impact of intron content of PT (%) on gene expression level is most noticeable in all three monocots. Furthermore, rice is found to be an intermediate if we consider the variation of gene components concerning gene expression between dicots and monocots (S2 and S3 Figs). Hence, we also analysed various properties of promoter regions associated with them for their relationship with various gene expression parameters in dicots and monocots to examine if differences also persist in these regions.

**Table 3:** Beta-coefficient values of arabidopsis, rice, sorghum and maize.

	Length of gene	Intron content	Number of intron	Length of 5’UTR	Length of 3'UTR	GC% of exon	GC% of Intron	GC% of 5'UTR	GC% of 3'UTR	AFE	Dnase 1	NPP	Curvature
***A*. *thaliana***													
b1	-0.09	0.18	0.01	0.1	0.31	0.11	0.17	0.03	-0.11	-0.07	-0.02	0.08	0.01
*p-* value	1.49E-14	5.85E-70	0.21*	9.61E-18	1.66E-69	4.71E-39	2.45E-86	5.11E-03	5.09E-11	5.06E-12	0.04*	4.31E-10	0.17*
b2	-0.35	0.05	0.09	0.11	0.28	0.22	-0.11	-0.09	-0.21	-0.04	0.07	0.04	0.01
*p-* value	9.30E-169	1.15E-07	7.14E-14	1.21E-18	1.06E-55	5.14E-146	4.53E-34	1.84E-13	4.65E-32	4.54E-05	5.87E-10	3.33E-03	0.64*
**b1/b2**	**0.26**	**3.22**	**0.16**	**0.94**	**1.08**	**0.48**	**-**	**-**	**0.53**	**1.89**	**0.31**	**2.05**	**2.77**
***O*. *sativa***													
b1	-0.05	0.24	-0.03	0.01	-0.005	-0.18	0.09	0.09	0.07	-0.11	-0.15	-0.02	0.002
*p-* value	4.41E-06	7.63E-141	1.44E-03	0.35*	0.75*	4.63E-69	8.28E-23	7.81E-21	3.08E-07	3.22E-33	2.12E-56	0.01	0.78*
b2	-0.25	0.2	0.04	0.05	-0.02	0.06	-0.02	0.04	0.09	-0.11	-0.01	0.006	-0.002
*p-* value	5.68E-99	8.35E-91	1.11E-03	3.73E-07	0.14*	7.31E-09	0.04*	6.21E-04	1.61E-08	3.12E-31	0.19*	0.51*	0.83*
**b1/b2**	**0.21**	**1.21**	**-**	**0.17**	**0.2**	**-**	**4.66**	**2.65**	**0.87**	**0.99**	**11.95**	**3.72**	**1.29**
***S*. *bicolor***													
b1	-0.05	0.17	0.03	0.08	0.15	-0.01	0.12	-	-	-0.07	-0.1	0.01	0.04
*p-* value	9.66E-06	3.22E-69	6.94E-03	3.80E-16	3.88E-51	0.13*	1.61E-42	-	-	3.01E-17	3.25E-29	0.02*	6.05E-05
b2	-0.08	0.12	0.08	0.19	0.15	-0.09	0.01	-	-	-0.04	-0.01	0.006	0.03
*p-* value	7.87E-13	4.42E-38	1.68E-19	7.56E-96	2.80E-51	6.56E-24	0.13*	-	-	7.20E-06	0.22*	0.47*	9.79E-04
**b1/b2**	**0.63**	**1.41**	**0.31**	**0.4**	**1.03**	**0.15**	**9.34**	**-**	**-**	**1.94**	**9.5**	**3.12**	**1.25**
***Z*. *mays***													
b1	0.13	0.13	-0.03	0.07	0.13	-0.34	-0.02	0.09	0.01	-0.05	-0.12	-0.001	0.001
*p-* value	1.89E-35	1.48E-47	5.49E-04	6.01E-10	8.26E-27	5.52E-292	4.70E-03	2.72E-17	0.43*	8.40E-12	5.08E-44	0.92*	0.87*
b2	0.09	0.03	0.06	0.04	0.09	-0.32	-0.06	0.11	0.03	-0.02	-0.06	0.01	0.01
*p-* value	9.79E-17	8.42E-03	8.89E-13	2.60E-03	1.22E-11	1.04E-241	4.54E-14	2.76E-21	6.20E-03	7.56E-03	1.04E-10	0.12*	0.14*
**b1/b2**	**1.42**	**5.22**	**-**	**1.94**	**1.49**	**1.04**	**0.35**	**0.84**	**0.27**	**2.42**	**2.04**	**0.05**	**0.1**

Beta-coefficient values of breath are presented as b1 and beta-coefficient of level as b2. R^2^ value for breadth and level in arabidopsis is 0.19 and 0.13, in rice 0.13 and 0.07, in sorghum 0.08 and 0.14 and in maize 0.34 and 0.27. Ratio of coefficient breadth/level (b1/b2), only for values with same sign are calculated and presented in bold. After Bonferroni corrections insignificant *p*-values are marked in *.

### Promoters of genes with varied expression have specific DNA structural features

**Fig 3:**
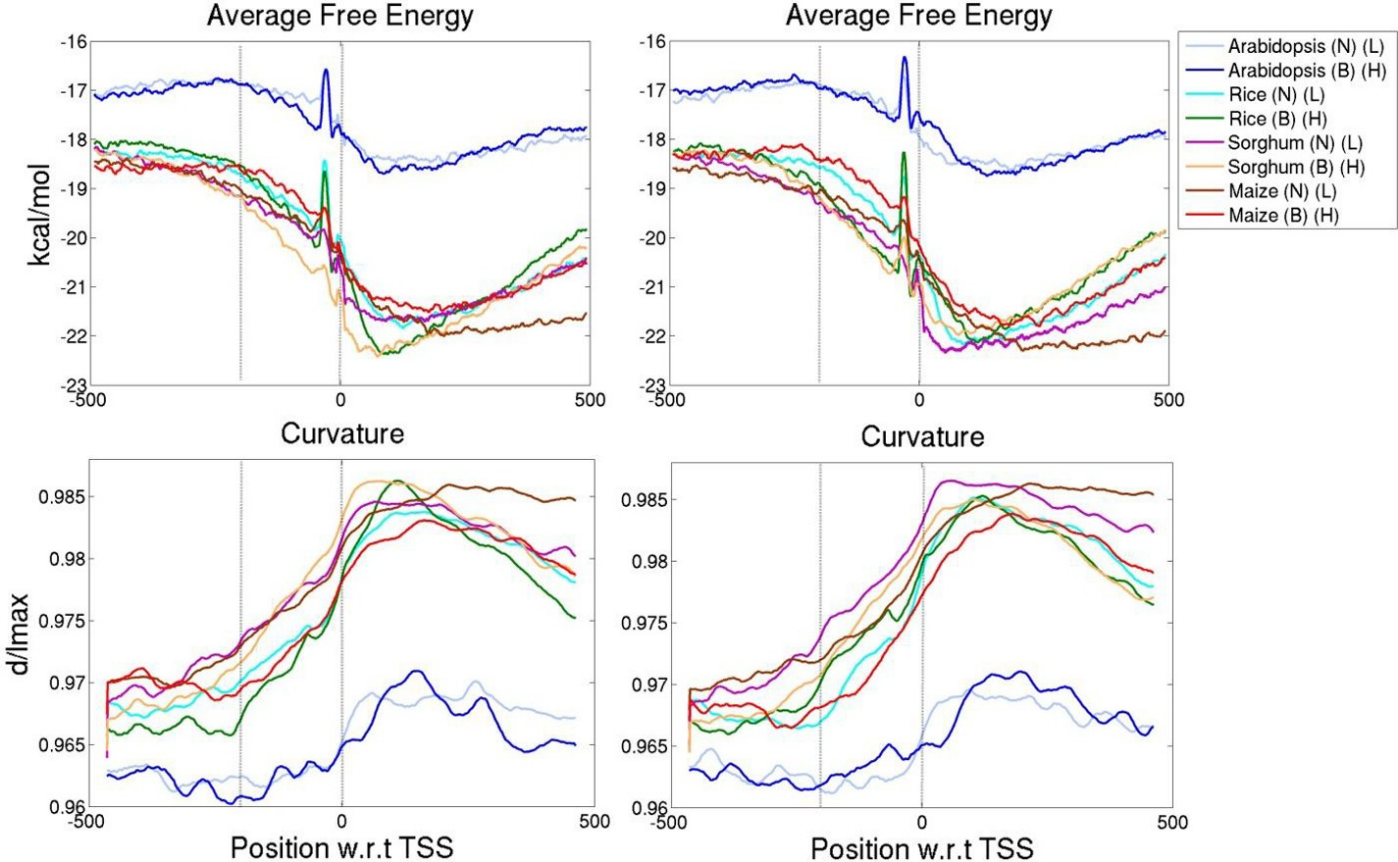
Structural properties of promoters (-500 nucleotides (nt) upstream to +500 nt downstream of TSS) of varying expression breadth (left panel) and level (right panel) are presented for arabidopsis, rice, sorghum and maize. Based on number of tissues in which a gene is expressed as well as average expression values, genes were sorted in increasing order from which top 10% and bottom 10% of genes are included in this analysis. DNA stability, a dinucleotide secondary structure property was represented by AFE (Average free energy) in kcal/mol. Distribution of curvature in the promoter region was calculated by using BMHT dinucleotide parameters. B stands for broadly expressed dataset and N for narrowly expressed genes, whereas H represents highly expressed and L lowly expressed dataset. ‘0’ on x-axis corresponds to the TSS position.

**Fig 4:**
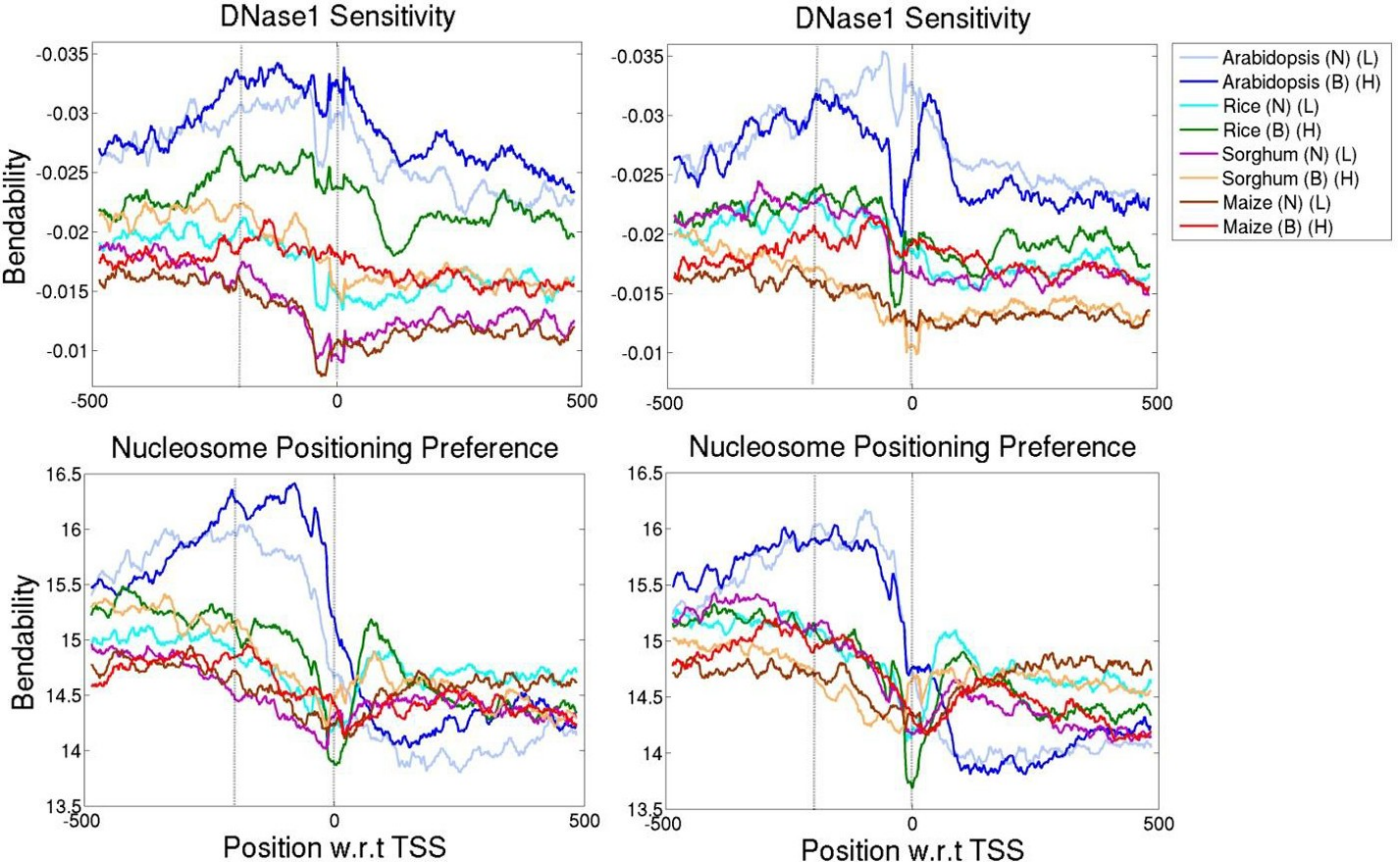
Bendability is plotted for the promoter region (-500 to +500) of top 10% and bottom 10% of the expression breadth (left panel) and level (right panel). Bendability or flexibility, trinucleotide secondary structural properties were measured by predicting DNaseI sensitivity and Nucleosome position preference (NPP). B stands for broadly expressed dataset and N narrowly expressed, whereas H represents highly expressed and L lowly expressed dataset.

Structural properties of the promoter modulate the gene expression [17, 18, 53, 54] by differential binding of transcription factors as well as by depletion of nucleosome formation [55]. Thus, to understand the difference in parameters influencing gene expression, promoter architecture has also been studied for all four plants. Four structural features, average free energy (AFE), DNase 1 sensitivity, nucleosome positioning preference (NPP) and curvature were calculated for the promoter regions of genes and correlated with change in their expression level and breadth. Genes were sorted based on the average expression value and number of expressed tissue samples and split into 10 equal bins, where each bin contains 10% of the data as described earlier. Structural properties were analysed for the promoter regions spanning upstream -500 to downstream +500 nucleotides with respect to the position of TSS (transcription start site). Averaged values of structural properties for genes lying in top and bottom 10% bins for average expression level and breadth were analysed (Figs 3 and 4).

The averaged DNA structural property plots indicate that promoter regions for all genes, narrowly and broadly expressed, as well as lowly and highly expressed genes, are less stable, less bendable and more curved in their promoter region as compared to flanking sequences. For all four structural properties, offbeat values are observed in -200 to 0 region upstream of TSS, which is demarcated by a vertical dotted line in Figs 3 and 4. The AFE plot illustrates that genomic sequences of arabidopsis are less stable than other plants (Fig 3). This stability difference between arabidopsis and rice has been previously reported and correlates with the difference in their GC content [47]. DNA stability is a measure of the ease of duplex melting, an essential process during transcription. Hence promoter regions being relatively unstable compared to flanking sequences in plants [47] as well as in other eukaryotes [56] facilitates transcription machinery to assemble and initiate DNA transcription. Interestingly maximum AFE difference was observed in the upstream region (-200 to 0) of narrowly and broadly expressed genes and also between highly and lowly expressed genes for all plants. Moreover, the differences persist even downstream of TSS in case of sorghum and rice (Fig 3).

Intrinsic curvature of promoter regions was also analysed and compared for the promoters of top and bottom 10% of expression breadth and level (Fig 3). Promoter regions are more curved in the vicinity of TSS compared to flanking sequences [17, 54]. Intrinsically curved DNA near the TSS also facilitates protein binding and transcription initiation [55]. Higher curvature of promoter sequences has been previously reported in prokaryotes as well as lower eukaryotes [17, 36, 53, 54]. When profiles of gene expression breadth and level genes were compared in detail, we found that differences in curvature property are more pronounced in genes which differ in their expression level as compared to those with different expression breadth. Curvature values of highly and lowly expressed genes are well separated in the promoter regions (-200 to 0) in all plants, while separation between broadly and narrowly expressed genes is clear only in maize.

Maximum difference in both bendability profiles was seen in promoter regions spanning -200 to 0 nucleotide upstream of TSS, as compared to the flanking sequences (Fig 4). A clear separation was observed in the vicinity of TSS between narrowly and broadly expressed gene promoter sequences. Here, we have analysed bendability by two trinucleotide based models; DNase 1 sensitivity and NPP. DNase 1 sensitivity model predicts the ease bending of trinucleotides towards major groove based on existing experimental data from DNase1 cutting frequency study [34]. Moreover NPP model measures the flexibility or rotational preference of trinucleotide around the histone core [35]. Basically, both models provide information regarding the bendability or flexibility of DNA sequence. Our results uncovered that promoter regions of broadly expressed genes are predicted to be less bendable by both the models. A striking difference was seen in DNase1 sensitivity between highly and lowly expressed genes of arabidopsis, although the difference is marginally less for other plants. Nevertheless, the difference in bendability predicted by both DNase1 and NPP, between top and bottom 10% of the promoters, is more prominent for expression breadth as compared to expression level. Less bendable or rigid structure of promoter helps in the formation of nucleosome free regions which facilitates the formation of transcription complex, while transcription of narrowly expressed or tissue specific genes is highly regulated by both proximal and distal promoters (enhancers) [57].

To quantify the significance of observed differences in structural properties of the promoter sequences from highly to lowly and from broadly to narrowly expressed genes, Kolmogorov-Smirnov (KS) nonparametric test was carried out. Two sample KS test was performed based on cumulative distribution functions to elucidate the difference in values of structural properties (S4 Fig). All property values are found to be significantly different at a *p* value ≤ 0.001 for promoter regions (-200 to 0) of top and bottom 10% of expression data. The cumulative distribution frequency plot shows that bendability (DNase1 sensitivity and NPP) has a similar trend for expression breadth in all plants, with broadly expressed genes being less bendable (S4 Fig). However, AFE showed an opposite trend for both expression level and breadth in maize, while curvature is unique in sorghum and maize for gene expression level.

#### Influence of structural properties on gene expression

**Fig 5:**
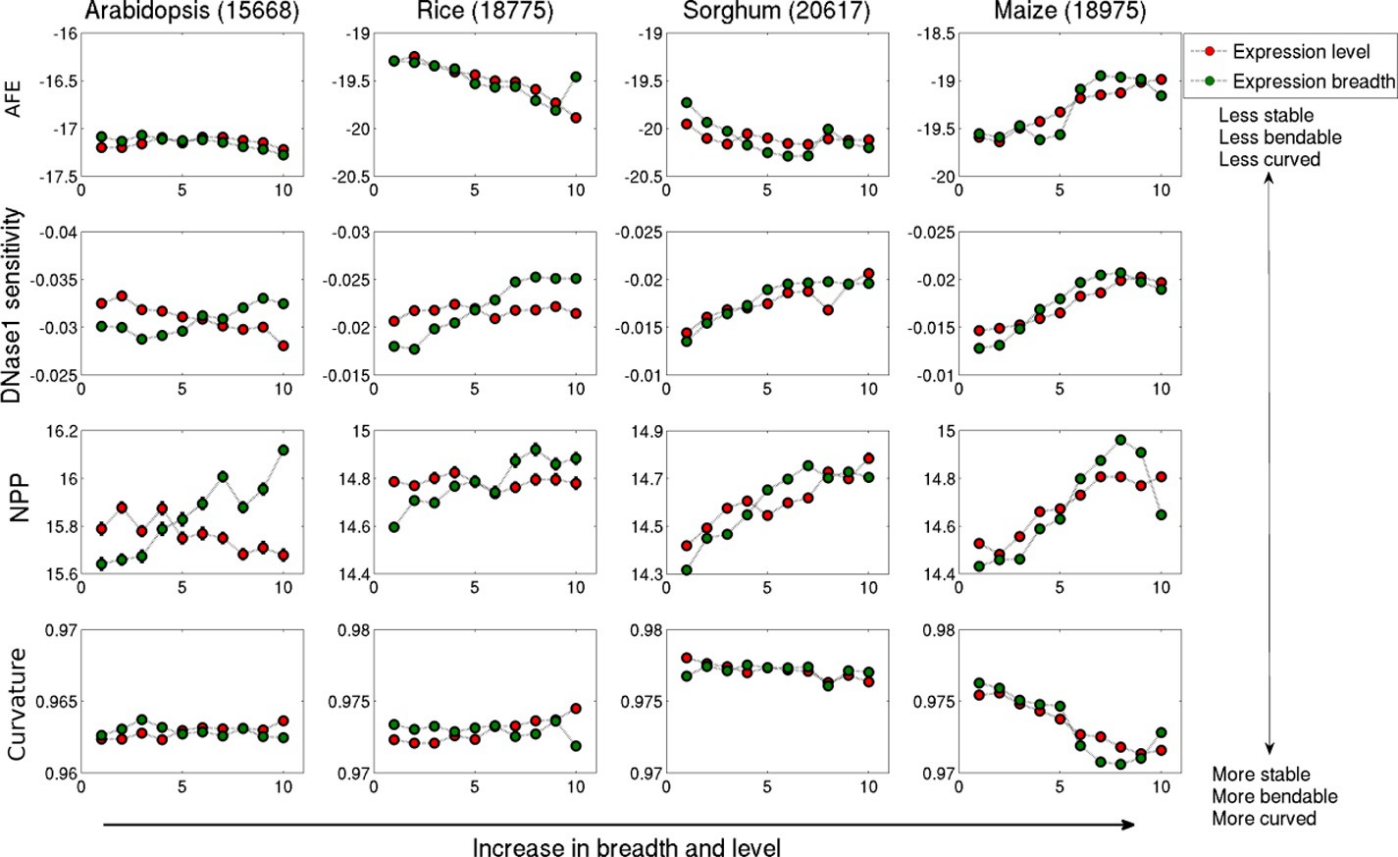
Structural feature parameter values for the promoter regions (-200 to 0) of arabidopsis, rice sorghum and maize. The averaged value of each 10 percentile bin for AFE, DNase 1 sensitivity, NPP and curvature are plotted. Genes were sorted based on the average expression value and split into 10 equal bins, where each bin contains 10% of the data. The bins are numbered 1 to 10 with increasing value of expression level and breadth.

In addition to the evaluation of promoter region in lowest and highest percentiles (top and bottom ten percent of the expression data), we have also examined the structural properties values for the entire range of expression values, for both breadth and level. Structural properties were analysed for the promoter regions of -200 to 0 for further analysis. Averaged values of corresponding structural properties for promoter regions were plotted from lowly to highly and from broadly to narrowly expressed genes (Fig 5). The profile of averaged free energy (AFE) values from narrow to broad expression showed a similar trend for rice and sorghum, which is opposite to that for maize, where promoter regions are less stable for broadly as well as highly expressed genes. In rice and sorghum, highly and broadly expressed genes are more stable while in arabidopsis, gene expression level does not show much variation with AFE (Fig 5).

Among all promoter properties, DNase 1 sensitivity and NPP showed linear correlation for expression breadth, while expression level is inversely correlated to bendability only in arabidopsis. Results showed that broadly expressed (constitutively expressed) genes are less bendable indicating that these rigid promoter regions cannot be easily wrapped around the histones to form nucleosome and are easily accessible to the transcription machinery to bind and transcribe the gene. Studies in yeast had also reported that the promoters of variably expressed genes are highly regulated and occupied with nucleosome while constitutively expressed genes have nucleosome excluded regions, suggesting rigidity in their promoter region [58]. A negative correlation is noticed between curvature and expression level as well as with breadth in maize. Hence, we hypothesize that promoter regions of plant genes with variable expression level and breadth have specific structural features.

### Multivariate multiple regression model

Our analysis of gene expression reveals that various genomic traits have different effects in the four plant systems. To arrive at a more quantitative relationship, we have built a multivariate multiple regression model [16] by combining genomic parameters and promoter properties. The various parameters are used as independent variable, whereas gene expression breadth and level are the dependent variables. Gene expression is found to be significantly and highly correlated with several traits in arabidopsis, rice, sorghum and maize, hence, initially we included all 17 parameters to generate the model. As multicollinearity would affect the model and also give an unnecessary rise in regression coefficient value [59], we have further analysed the variance inflation factors (VIFs) [60] (see Materials and Methods).

The parameters, length of exon and intron are removed from the parameters related to gene compactness because of their high VIF values (S1 Table). Intron length and exon length show high correlation with gene length and GC% of exon respectively [61]. We also removed GC% of PT and difference in GC% between exon and intron, since both of them are correlated with GC% of exon. Totally 13 parameters were used as independent variables to build the model in arabidopsis, rice and maize (S1 Table). Only 11 parameters were included for sorghum, since the GC% of 5’- and 3’ UTRs shows a high correlation with the length of UTRs, these features were excluded.

### Relative effect of different traits on level and breadth

**Fig 6:**
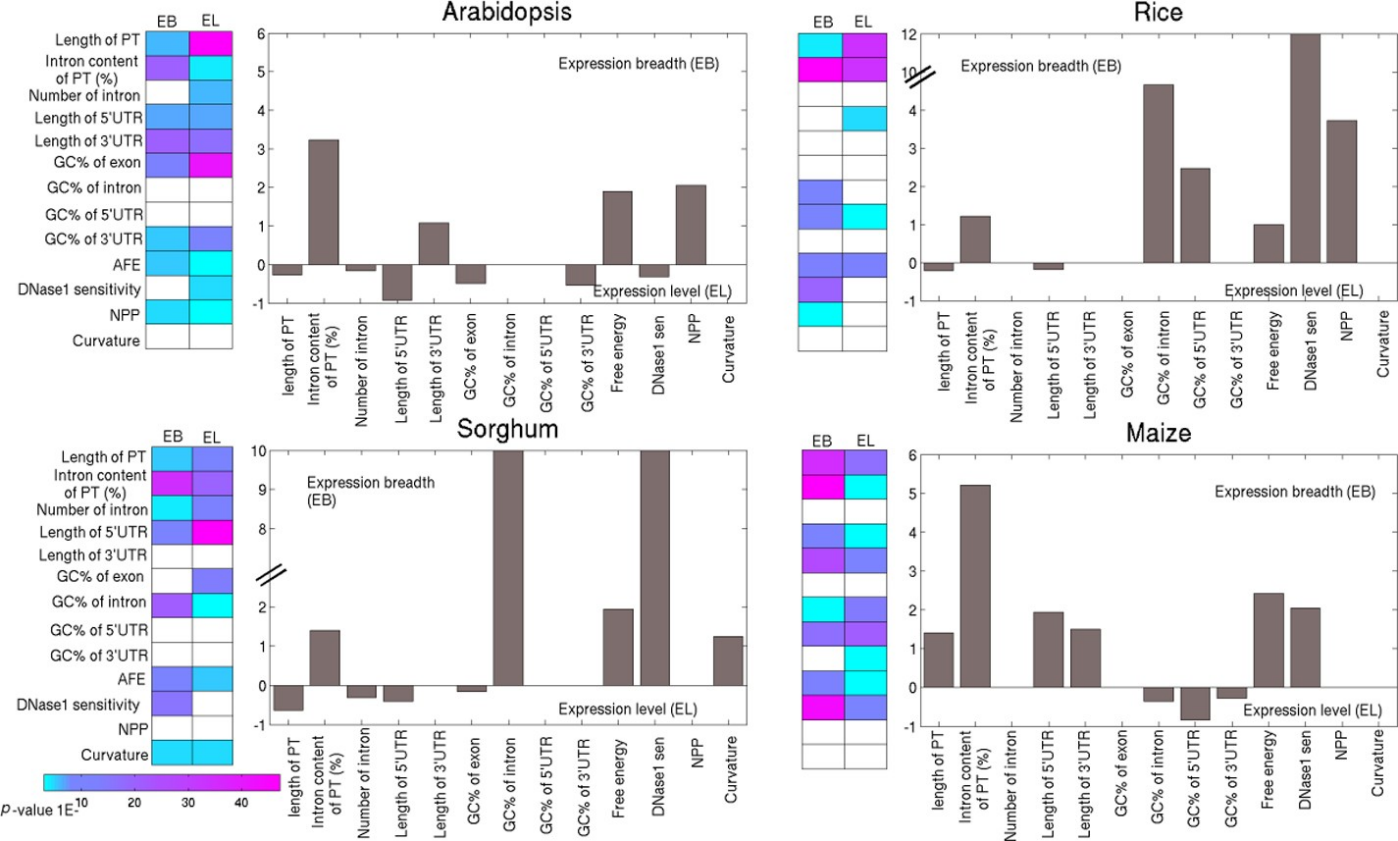
The influence of parameters on expression level or breadth is plotted for arabidopsis, rice, sorghum and maize. Ratios of the coefficient b1/b2 (from Table 3) are plotted only for significant *p*-values of either of the measures (breadth/level) with same sign. Corresponding *p*-values for breadth and level individually are shown on left of each plot with values increasing from cyan to magenta color (the empty color bar represents insignificant *p*-values).

Multivariate multiple regression models generated on plants predicted 7 to 34% change in gene expression level and breadth that can be attributed to the studied independent variable. Model explains the variation in breadth and level (as indicated by respective R square values) to be 19 and 13% in arabidopsis, in rice as 13 and 7%, in sorghum as 8 and 14% and in maize as 35 and 27% (Table 3). Effect on a dependent variable (level and breath) by common independent variables can be compared by its coefficient values; however, an estimate of the level of significance in terms of *p*-values can give a better quantitative idea. Our analysis includes multiple parameters, therefore Bonferroni correction is used to evaluate significant *p*-values for multiple testing [42]. The dependent variables were normalized to make the mean value as 0 and variance as 1 so that they can be directly compared. Furthermore, we compared the coefficient of breadth and level for each independent variable by calculating the ratio of coefficient of breadth and level. Dependent variables are also compared for their respective *p*-values. Only values with similar direction of coefficient (i.e. both level and breadth are positive or negative) are considered to compare the relative strength between the expression parameters. Moreover, if one of the traits is found to be insignificant, then only the significant one was included in the analysis. In addition to that, we have removed those cases where breadth/level ratio is 1. The strength of gene expression parameters and their respective *p*-values are plotted in Fig 6.

Regression analysis reveals that gene compactness in terms of the length of PT is found to be significantly negatively correlated in arabidopsis, rice and sorghum for both the expression parameters, with breadth being 0.2 to 0.6 times less affected as compared to level (Fig 6). These results are in agreement with analysis reported earlier in arabidopsis and rice as well as in human and mouse [6, 62] and a possible explanation could be the evolutionary conservation of the broadly and highly expressed short genes. Among all properties related to compactness, intron content is positively correlated with both the traits but more strongly related to expression breadth and these results were found to be consistent for all four plant systems. The ratio of breadth/level in rice maize is found to vary from 1.2 times to 5.2 times stronger than level. These results agree with the earlier study on gene density and expression level/breadth in arabidopsis and human [5, 48]. In all studied plant systems, number of introns shows positive correlation with expression level yet found to be stronger for level only in arabidopsis and sorghum by regression model (Table 3A and 3C). Broadly expressed genes are intron depleted in rice and maize while contradictory results was seen in rice by Yang 2009, suggesting that in plants this genomic trait plays distinct role on expression components [6]. In comparison to intron, lengths of 3’ UTR and 5’ UTR are positively correlated to both level and breadth in most of the plants and our analysis also reveals that length of 5’ UTR has a strong effect on gene expression level (Fig 6) in arabidopsis, rice and sorghum. Long intron in 5’ UTR plays a critical role in gene expression level which is reported earlier for arabidopsis [63]. Furthermore, relationship between GC composition of the intron, exon, 5’ and 3’ UTR with expression level and breadth is quite variable. The diverse nature of GC% of intron with level and breadth has also been reported in human and mouse [16]. Summarising the above results from regression analysis, we found that among parameters related to the gene compactness, intron content is more strongly related to breadth of expression and is observed commonly in plants, while the lengths of PT and 5’ UTR are better measures of gene expression level in arabidopsis, rice and sorghum.

Promoter properties are also analysed with the variation of expression parameters (level and breadth) by multivariate analysis. Free energy of the promoter region is negatively linked to both level and breadth, being observed in all four plants. Interestingly, in three of the studied plants, with only rice being an exception, breadth shows stronger correlation, approximately 2-fold stronger than level, conveying that broadly expressed genes are generally more stable. Among the bendability properties, DNase 1 sensitivity appears to strongly govern gene expression breadth in monocots and gene expression level in dicots (Fig 6). These results provide an insight into the evolutionary changes favoured in the promoter regions in dicot and monocot, which had diverged around 150 million years ago [64]. The multivariate regression analysis has revealed whether expression level or breadth is more strongly influenced by various genomic parameters. It suggests that breadth can be quantified by gene compactness, linked with intron content of PT (%) and promoter properties such as free energy. Our rigorous regression analysis on plants has come up with two major findings: 1. Broadly expressed genes are less compact in nature and 2. Promoters of broadly expressed genes are stable in plants.

#### 1. Broadly expressed genes are less compact in nature

Introns play diversified roles in eukaryotes [65] such as initiation of transcription, stable expression, genomic design, alternative splicing, etc. Regression analysis confirms that the intron content of PT (%) is positively linked to both expression level and breadth (Fig 6), but expression breadth is more strongly affected than gene expression level, suggesting that intron density is higher in broadly expressed genes. Earlier studies in human and arabidopsis revealed that intron density is positively linked to both expression level and breadth [5, 6, 48, 49]. The positive correlation of intron density with gene expression parameters implies the biasness towards the intron gain for evolutionarily conserved genes for broadly expressed as well as highly expressed genes while an opposite scenario is spotted in *C*. *elegans* [50]. However, in mammals, gene compactness is more strongly negatively related to expression level than breadth [16, 49].

It has also been suggested that introns are associated with nucleosome disfavouring regions which help in nucleosome phasing in exonic region [66], thus acting as regulatory elements for gene expression. Regulatory function of intron could give a better justification for their presence in broadly expressed and highly expressed genes of plants [65, 67]. Moreover, a comparative study on human intron-less and intron-containing genes has revealed that intron-less genes are associated with low expression and are tissue specific [68]. Regulation of gene expression can be modulated by introns and spliceosomes during transcription by polyadenylation [69, 70]. In addition to that, broadly expressed genes are positively regulated by alternative splicing in human [71] which supports our finding, and suggests that introns play a crucial role in the regulation of gene expression.

#### 2. Promoters of broadly expressed genes are stable in plants

Present multiple regression analysis on promoter properties revealed that free energy is negatively correlated to both gene expression level and breadth with breadth showing a stronger correlation. Free energy is essentially a measure of stability based on dinucleotide parameters. The direct relationship of free energy and AT content has been reported earlier [47]. Promoter regions are less stable and AT- rich compared to its flanking sequence in plants, which is quite clear from the Fig 3. Hence free energy can be related to AT content of the promoter regions. Regression analysis illustrated that promoters of narrowly expressed (tissue specific) genes are less stable or AT-rich, while broadly expressed genes as well as highly expressed genes are GC-rich.

It is known that expression of genes is determined by the nucleosome occupancy in the promoter as well as in the gene body. Nucleosome occupancy can be influenced either by the nucleotide composition of the promoter, the intrinsic property of DNA sequence [72, 73] or by the availability of the transcription factor that recognizes the *cis*-motif signal. An extensive study on human and yeast depicted the enrichment of poly(dA/dT) region in the nucleosome-depleted regions (NDR), generally found near the TSS [74]. An opposite scenario is reported for the depletion of the nucleosome in plants; especially in arabidopsis and rice, NDRs have higher G/C content [75, 76]. The pattern of nucleosome distribution around TSS and gene bodies determines the gene expression level and specificity in arabidopsis and maize which is characteristically different from that in animals [77, 78]. This indicates that in plants, promoter region of genes with ubiquitous expression are GC-rich, which strongly suggests presence of NDR in the promoter region. Studies on the NDR region and ubiquitous expression also support our results, however unfavourable interaction of histone and promoter sequence in NDR is not by itself sufficient, because the presence of chromatin remodelers (Nucleosome depletion factors) [79, 80] and nucleosome availability depends on the condition studied [81].

In addition, results of regression analysis illustrated the inverse relationship of DNase 1 sensitivity with expression parameters of dicot and monocot promoters which led us to examine the compositional difference in their promoter regions.

### Hexamer frequency distribution differs in promoter regions of narrowly and broadly expressed genes

**Fig 7:**
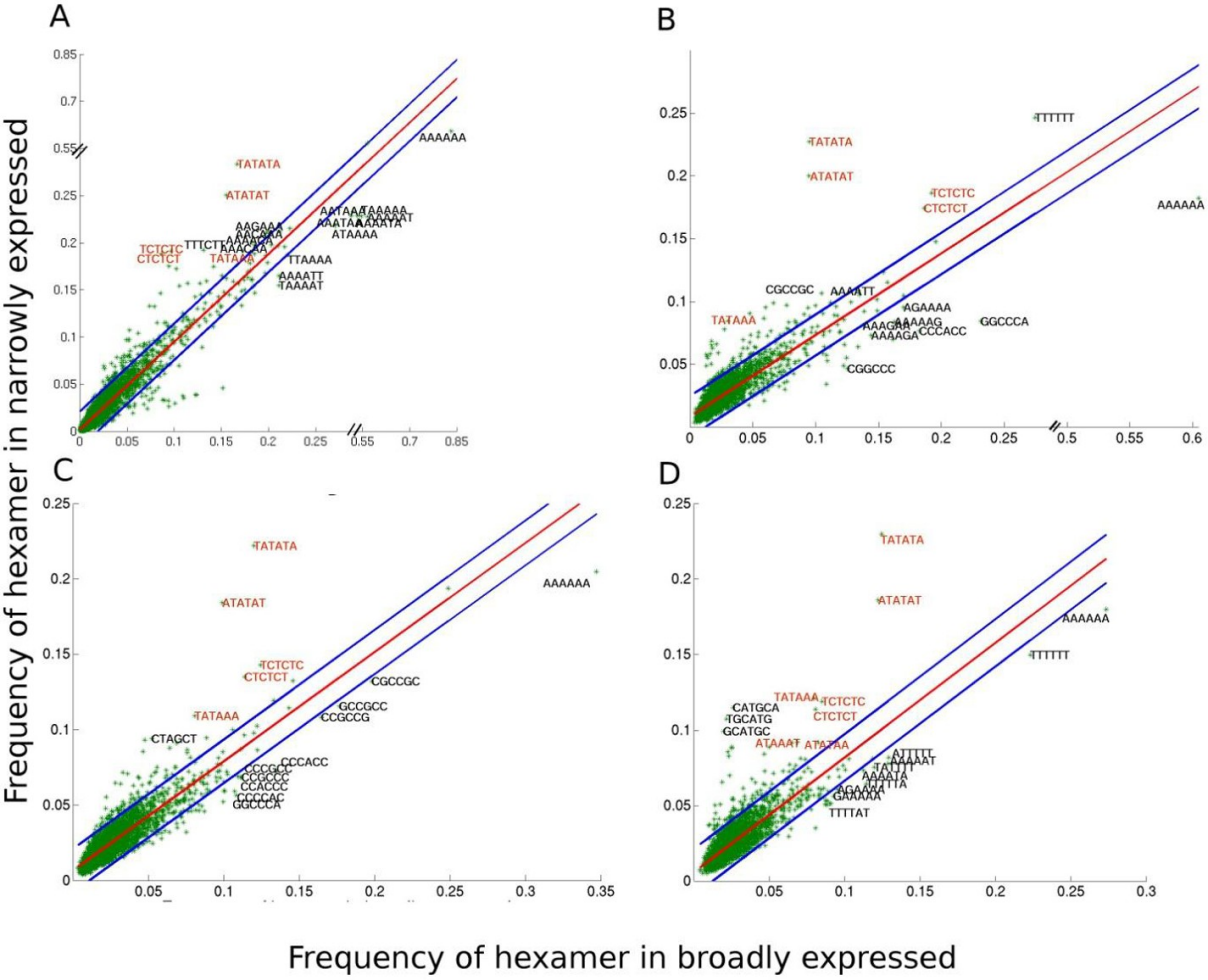
**Hexamer distribution plot shows the preferred hexamers in the promoter regions (-200 to 0) of broadly and narrowly expressed genes of arabidopsis (A), rice (B), sorghum (C) and maize (D).** All possible hexamers of 10% broadly and narrowly expressed genes are plotted with red line as best fit line and blue lines are the 2σ deviated from best fit. Top ten enriched hexamers of broadly and narrowly expressed sets from 2σ deviated dataset are labelled here. Hexamers that match with the consensus motif of TATA and Y-patch are shown in red color.

Various regulatory motifs such as TATA box, CCAAT box [82, 83], GC box [84] and many more transcription factor binding sites (TFBs), are found commonly in plants and animals and they play an important role in the initiation and regulation of transcription. These motifs are mostly present in the promoter regions in the vicinity of TSS. Hence we have investigated as to which hexamers are favoured in the promoter region (-200 to 0) of narrowly and broadly expressed genes (Fig 7). Here we have presented only the top ten hexamers which are > 2σ deviated from the best fit line (see in materials and methods). For all plants at least twice the numbers of hexamers are 2σ deviated in narrowly expressed genes as compared to broadly expressed genes. Moreover, interestingly GC-rich hexamers are overrepresented in the promoter regions of broadly expressed set of rice and sorghum which is further supported by the enrichment of GC-rich trimer (S5 Fig). GC-rich trimers were enriched in the promoter regions of broadly expressed dataset of rice and sorghum which was confirmed from trimer frequency plot (S5 Fig). Difference in frequency of trimers in promoter regions spanning from -200 to 0 between broadly and narrowly expressed genes were plotted for both the bendability models (DNase 1 sensitivity and Nucleosome positioning preference) showed the GC-richness in the promoters of constitutively expressed genes of sorghum and rice (See materials and methods).

In the current study, consensus sequence of TATA box and Y-patch consisting of T/C-rich motifs were preferentially found in narrowly expressed set of all plants and these motifs have been well studied previously in rice and arabidopsis [85–88] (Fig 7). The advancement in genomics studies has given a new perspective regarding the presence of TATA-box in the core promoter. In eukaryotes like human and yeast, less than 20% of promoters contain TATA-box [89, 90] whereas TATA box containing promoters contribute less than 39% in arabidopsis [91], 19% in rice [85] and 38% in maize [92] harbour TATA-box. Similarly, Y-patch which is restricted to plant promoters is spotted in less than 18% promoters in arabidopsis and 50% in rice [85, 91]. Moreover, enrichment of these motifs in promoter regions of tissue specific genes is also supported by previous studies [91, 93]. Our results revealed many motifs in tissue specific (narrowly expressed) genes for TF binding sites associated with biotic and abiotic stress response genes, as well as hormonal stimulations in the specific tissue. The transcriptional machinery formed on TATA-box by itself can give basal level of transcription [86] however *cis*-element in a combinatorial mode with regulatory TFs facilitates gene regulation and expression at maximal level [94]. TATA-containing genes are associated with stress related genes whereas TATA-less genes aid ubiquitous expression [95, 96]. Thus, presence of TATA-motif and various regulatory motifs associated with stress response in narrowly expressed genes is quite understandable. Now to understand the evolutionary relationship among the promoter regions as well as in the genic region, an extensive study on the orthologous group was performed to expose the shared molecular pathways.

### Variation in expression parameters along the gene copy number in plants

Duplication of genes provides an important base in adaptations for plants and animals during evolution [97]. Functional divergence plays a major role in the evolution of duplication of genes as well as genomes, considering the augmented phenotypic plasticity in the organism [98]. Change in copy number of gene by gene duplication has a drastic and divergent effect on gene expression in plants that could give rise to new phenotypes and divergent gene expression is mostly regarded as a maker of divergent gene function [20]. The genome and gene duplication event are observed frequently in angiosperms. Arabidopsis and rice are two wonderful models that have experienced gene and genome duplications, which helped them in evolving new species as well as homologous pairs [99–101]. Moreover, the potentiality of duplicate genes and distinguishing character of singletons genes have been studied extensively in rice [102]. In this study, orthologous genes are searched in all four plants to analyse the relationship between gene copy number and gene expression measure.

**Fig 8:**
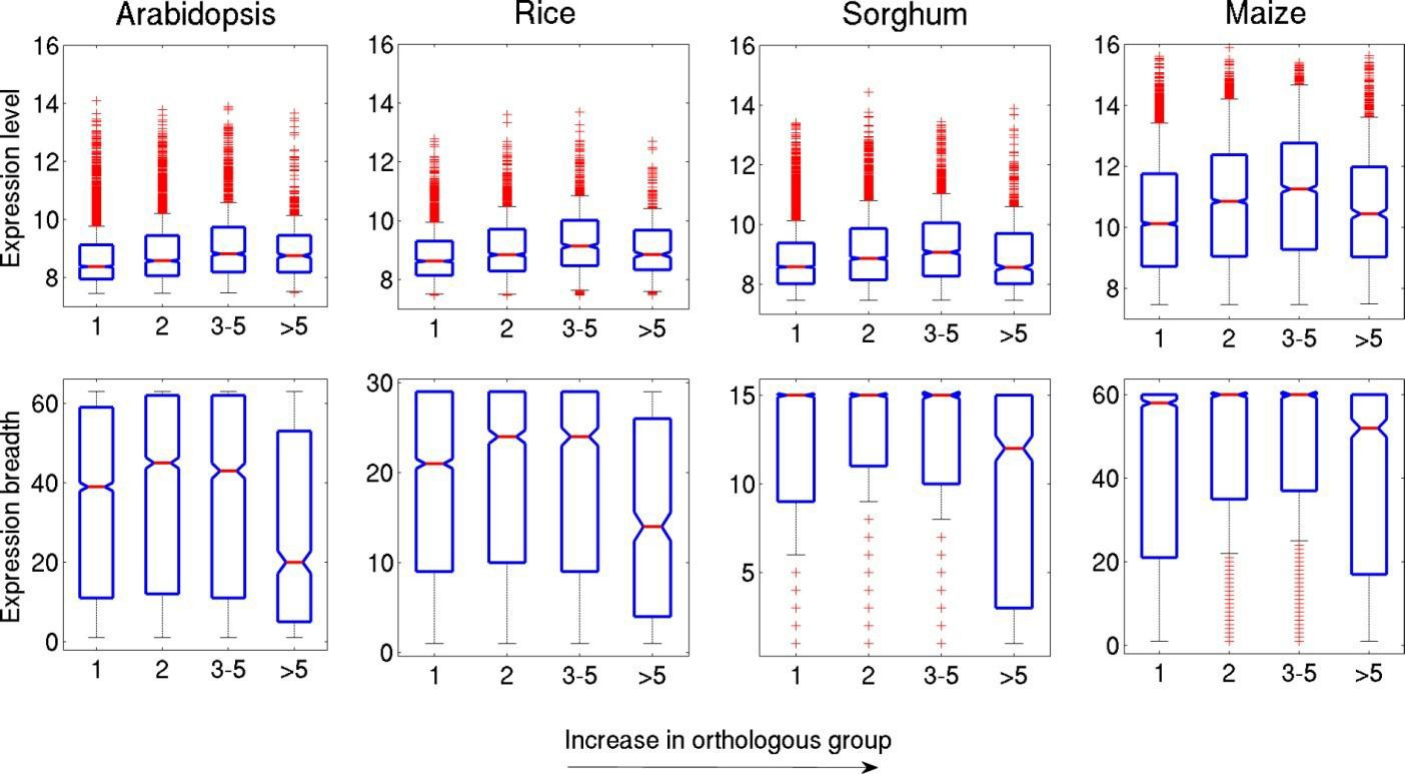
The relationship between size of orthologous group and expression level and expression breadth. Top and bottom spaces of the box are 25th and 75th percentiles of the dataset. Here the mid line of the box represents the median position and the outliers are presented in red color.

We used rice as reference genomes to cluster orthologous groups in other genomes and identified singleton or single copy, 3 to 5 copy number and >5 copy number genes (see the Materials and Methods). Single copy gene dataset consists of 4828, 3334, 9031 and 6242 number of genes in arabidopsis, rice, sorghum and maize respectively while the smallest datasets are for >5 copy number genes. As compared to multiple copy genes, higher numbers of singleton genes are observed from earlier study in arabidopsis, rice and sorghum [21]. In this study, we observed significant correlation between gene expression level and size of orthologous group (Fig 8). Single copy genes were lowly expressed compared to multi copy number of genes (*p* < 0.001, two-sided Mann-Whitney U test) while singleton genes are broadly expressed and are significantly different from multi copy number of genes (*p* < 0.001, two-sided Mann-Whitney U test). Multi copy number genes are tissue specific. Similar results were also found for gymnosperm [22] suggesting that tissue specific genes are commonly present in multi copy number to perform a highly regulated function.

#### A comparative study of structural properties of promoters of duplicate genes across plant species

Increase in orthologous group and the changes occurring in the four structural parameters of promoter regions (upstream -200 to 0 with respect to TSS) have also been studied. Structural properties of similar copy number genes across different plant species showed significant differences among them (S6 Fig). Stability (Free energy) profiles of the promoter regions are found to be different for sorghum and arabidopsis when they were clustered based on their gene copy number whereas in maize and rice they were very similarly placed. Promoter sequence of dicot plant (arabidopsis) is less stable than that of monocots [47]. As described by Morey et al., free energy profile reflects the AT content of the promoter region [47]. Thus we can conclude that promoter region of arabidopsis is AT-rich among the studied plants and promoters of sorghum are GC-rich. Like free energy, curvature study on promoters also shows a similar profile for rice and maize. Promoter sequences of arabidopsis are less stable and more curved compared to rice, maize and sorghum. Overall, the bendability (DNase 1 sensitivity and nucleosome position preference (NPP)) of promoter sequence of dicot showed a significant difference from monocots and found to be less bendable (S6 Fig).

The above comparative study on structural properties has revealed the evolutionary patterns, particularly between monocot and dicot and offers important insights into the promoter evolution in plants. The similar pattern of promoter properties for duplicate genes can be attributed to several factors. Increase in duplicate copy number of genes within a species could happen due to large scale event like whole-genome duplications (WGD), by segmentation or by tandem (unequal crossing over of chromosome), where the identical promoter is mostly preserved in the newly evolving gene as the parental gene [20, 103, 104]. Further, a series of genomic events on a set of duplicate genes could have generated a new promoter. For example, duplicate genes created by retrotransposons may undergo small substitutional changes compelled by natural selection [105–107]. These facts indicate that the relationships between gene duplication modes and promoter evolution are not clear and future efforts are necessary to arrive at a clear picture of changes occurring in promoters during gene duplications.

#### Variation of gene parameters with increase in orthologous groups

As a result of gene duplication, a significant change in gene parameters of the newly generated duplicate genes plays a central role in determining the divergent functions. Notably, changes in ratio of coding and noncoding regions in gene architecture help in adaptations of duplicate gene by providing a novel function to it [108–110]. Thus, in this study, we have further explored the variation of length and G+C content of PT, coding and noncoding region with increase in gene copy numbers. Interestingly, the length parameters like the length of PT, exon, 5’ UTR and number of intron showed a significant positive relationship with the gene copy number (S7 Fig). The increase in duplicate copy numbers in the gene is favoured by adding up nucleotides to exon and 5’ UTR region as well as to the introns. Interestingly these features are common in all plants studied so far. Nonetheless, the length parameter, intron content of the primary transcript (%) is declining for high copy number genes suggesting that the introns added to the duplicate genes are small in length. The above result implies that increase in intron number has increased the probability of different splice variants from the same transcript and hence increases the functional divergence [111]. Fate of duplicate genes, upon its survival, depends on the production of functional protein. New functional protein has an altered amino acid composition from the parental protein which is basically originated by modifications in exon-intron structure [66, 112]. Earlier study on arabidopsis have reported that duplicate genes are associated with long exon [112]. Our previous result (Fig 8) revealed that duplicate genes or multi copy number genes are tissue specific. Thus, we can link these two results and conclude that duplicate genes are longer in length (gene length/exon length) and are tissue specific in plants. However, the inclusion of small intron can be explained as they are a disfavoured region for nucleosome formation and spotting of exons by nucleosomes plays an important role in maintaining the exon and intron architecture of a gene [66].

The 5’ UTR and 3’ UTR are known to play a regulatory role in post-transcriptional process by increasing the mRNA stability, providing suitable localization, and translational efficiency. Moreover the increase in length of 5’ UTR in the duplicate genes (S7 Fig) has been explained by their nucleosome occupancy [113]. It is observed in yeast that a long 5’ UTR region can avoid the +1 nucleosome occupancy, which can play a regulatory role in gene expression. Also, the context of 5’ UTR sequence determines the gene expression as well as translational efficiency in yeast and in arabidopsis [114, 115]. Moreover, the presence of intron in 5’ UTR region has been documented by several groups and especially in arabidopsis, gene expression of EF1-A3 gene is enhanced by the existence of a long intron in the 5’UTR region [63, 116]. The above observation on effect of long 5’ UTR region on gene regulation can be indirectly correlated to increase in length of the gene and regulatory element in duplicate genes which are mostly tissue specific in nature (Fig 8).

Apart from length, GC content also plays an important part in gene expression. Yet, in all studied plant system, we could not find any clear divergence in the GC content of coding and noncoding region of duplicate genes except the decrease of GC% of 5’ UTR (S8 Fig). Though earlier studies on picea (gymnosperm) established a positive relationship between GC% of coding region and size of the gene family, we did not notice any such relationship in angiosperm [22].

### Functional pathways of varying expression breadth and gene copy number

**Fig 9:**
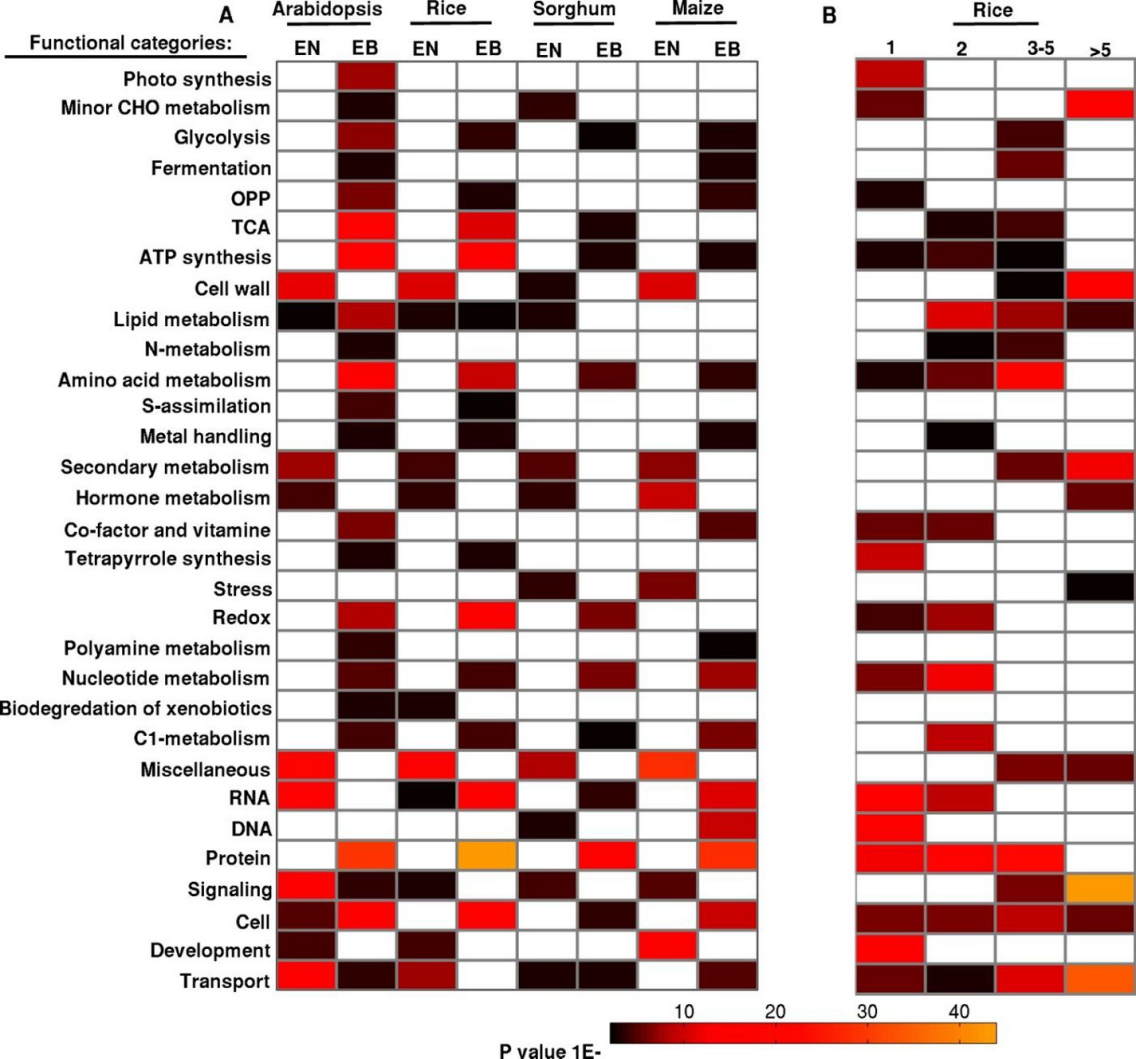
Gene Ontology (GO) enrichment is represented for broadly and narrowly expressed genes. (A) GO terms of broadly expressed (EB) and narrowly expressed (EN) genes are shown for arabidopsis, rice, sorghum and maize. (B) GO term enrichment for biological processes is shown for different size orthologous groups of rice. The functional groups are represented in colors ranging from black through red to yellow based on their increasing significance.

Functional categories of narrowly and broadly expressed datasets were analysed using MapMan [117]. Enriched functional categories are illustrated by using GO-MAPMAN (see the methods). The top and bottom 25% of the expression breadth data are taken to define ‘narrowly’ and ‘broadly’ expressed gene data set respectively and arranged in increasing order. GO terms augmented in both the datasets are presented for all four studied plants (Fig 9A). Overall, functional categories associated with glycolysis, ATP synthesis, amino acid metabolism, nucleotide metabolism and c1 metabolism are enriched for broadly/constitutively expressed genes (Fig 9A). These metabolisms are regarded as most constitutive systems which are necessary for anticipating the intra- and extracellular environment necessary to regulate the metabolism. However, GO terms related to cell wall, stress, hormone metabolism, secondary metabolism and development are enriched in tissue specific dataset. This result indicates that all of the above specific functions are confined to a few tissues and to particular environmental conditions.

Moreover, we also explored the enrichment of the same set of functional categories for different copy number genes in rice (Fig 9B). In our orthologous grouping, rice has been used as reference genome to search for duplicate genes in other plant systems; hence analysis of GO term of varying gene copy of rice can represent functional categories in remaining plant systems (arabidopsis, sorghum and maize). Analysis of GO term revealed that multi copy genes are related to stress, secondary metabolism, hormone metabolism and signalling pathways. As multi copy genes are found to be tissue specific (Fig 8), we can draw a rough conclusion that genes which are present in multiple copy number perform specific functions, respond to the change in environment and that these genes have evolved and accumulated in multiple copies in the genome during evolution, which helps them in further adaptation.

## Conclusion

Comprehensive analysis of gene expression variability of four different plant systems helps elucidate the common and variable features of gene architecture and promoter properties. Results of this study indicate a clear influence of gene architecture on expression level and breadth. Earlier studies have also strongly supported the notion that both coding and noncoding regions play a crucial role in the regulation of gene expression. From the multivariate multiple regression analysis reported here, it was inferred that the intron density is an important measure which regulates the breadth of gene expression in plants while other parameters related to gene architecture correlate diversely with the expression parameters and are found to be species specific. Thus, we concluded that broadly expressed genes are less compact and hypothesized that introns are the regulatory element favoured during evolution in broadly expressed genes. Similarly, promoter structural properties are known to play a crucial role in gene expression and our regression analysis showed that AFE (stability) values of promoter sequences are strong determinant of expression breadth. Though, free energy was negatively correlated to both expression level and breadth, promoters of narrowly expressed genes are found to be less stable due to being more AT rich as compared to promoters of broadly expressed genes. Moreover, a bendability profile comparison reveals that promoter regions of broadly expressed genes are less bendable, which is attributed to their being enriched in GC-rich motifs, a feature found to be common in plants.

This study also reveals that in plants, promoter regions of narrowly expressed genes have an abundance of TATA-motifs and Y patches, which might be linked with a more complex regulation mechanism to modulate gene expression in a specific tissue. In addition, this study also highlights the relationship between the length parameters of genes and gene copy number in plants. Finally, a broad conclusion can be drawn from our study that multi-copy orthologous genes in plants are long, highly regulated and tissue specific. However, detailed experimental studies on differences in promoters as well as gene architecture between parental and duplicate genes are required to uncover the evolution of duplicate genes in plant.

## Supporting information

S1 Fig Distribution of expression intensity of probe represented on log2 scale. Expression intensity of probe represented on log2 scale (A). Only genes with expression intensity >200 or >7.64 are included for further analysis. (B) The distribution of gene datasets in four plants. A gene is considered as expressed if the expression intensity value is >7.64 (on log2 scale). Histograms are plotted with bin size 1 for both expression level and expression breadth presented in green and blue color respectively.
(TIF)

S2 Fig The relationship between 7 different parameters of plant genes and their expression. The panel shows the mean of respective parameter values versus the average expression value in 1%, 5%, 10%, 15%, 20%, 25%, 30%, 35%, 40%, 45% and 50% quantiles for both sides of the whole data set in arabidopsis, rice, sorghum and maize. Intron-less genes are removed from this analysis.
(TIF)

S3 Fig The relationship between 6 different parameters of plant genes and their expression. The panel shows the mean of respective parameter values versus the average expression value in 1%, 5%, 10%, 15%, 20%, 25%, 30%, 35%, 40%, 45% and 50% quantiles for both sides of the whole data set in arabidopsis, rice, sorghum and maize. Intron-less genes are removed from this analysis.
(TIF)

S4 Fig Distributions of structural properties of DNA are illustrated by cumulative distribution function plot in the promoter regions spanning -200 to 0 with respect to TSS. All four properties are presented for arabidopsis, rice, sorghum and maize. The y-axis represents cumulative frequency (value ranges from 0 to 1) and x-axis denotes the values of structural properties.
(TIF)

S5 Fig Bar plot shows the difference in frequency of trimers in the promoter regions (-200 to 0) of broadly and narrowly expressed genes. Trimers of four plants are presented in four distinct colors. The top figure shows trimers sorted in order to their flexibility, from high (left) to low (right) for DNase 1 senitivity and the bottom one is for Nucleosome positioning preference sorted in accordance from the minor groove (left) to the major groove (right) with those showing no preference in the middle. Bar present on the positive side correspond to high trimer occurrence in broadly expressed genes.
(TIF)

S6 Fig Cumulative distribution function (CDF) plots for structural properties of the promoter regions spanning -200 to 0 nt with respect to TSS. All four properties are presented with an increase in the gene copy number of arabidopsis, rice, sorghum and maize. Similar copy number genes are grouped together and structural properties are calculated for specific plant, re-spective CDFs are juxtaposed for four different plants. The y-axis represents cumulative frequency (values ranges from 0 to 1) and x-axis denotes the values of various structural properties examined.
(TIF)

S7 Fig Seven different gene parameters related to the length of PT, coding and noncoding regions are plotted with an increase in the orthologous group. Top and bottom spaces of the box are 25th and 75th percentiles of the dataset with the mid line of the box represent the median position. Outliers are not shown here; nevertheless, these are included during the plotting of box plot. The significant positive relationship between length parameters and orthologous groups are shaded in yellow color.
(TIF)

S8 Fig Six different gene parameters related to G+C content of PT, coding and noncoding regions are plotted with an increase in the orthologous group. Top and bottom spaces of the box are 25th and 75th percentiles of the dataset with the mid line of the box represents the median position. Outliers are not shown here; nevertheless, these are included during the plotting of box plot. The significant negative relationship between length parameter and orthologous group is shaded in yellow color.
(TIF)

S1 Table VIF values of parameters after removal of highly correlated gene components. After removal of highly correlated gene components with other gene components such as length of intron, length of exon, GC% of PT and difference in GC% of exon and intron. GC% of 5’ UTR and 3’ UTR are removed from Sorghum to reduce the value of VIF of both the respective length of UTRs from 9.6 to 1.7 and 22.0 to 1.9 respectively.
(PDF)

S2 Table Gene families were grouped based on their orthologous copy in the genome. Rice was used as the reference genome to search the single copy, two copies, three to five copies and more than five genes in the queried genome.
(PDF)

S3 Table Statistics of parameters of 25% of lowly and highly expressed genes. Mean values of parameters are represented with the ± standard deviation of both sides of the data set in arabidopsis, rice, sorghum and maize. Zero intron genes are excluded from the analysis of parameters such as, Intron content of PT (%), length of intron, number of intron, GC% of intron and difference in GC% of intron and exon.
(PDF)
