# Supplementary figures and images for: Variation of gene expression in plants is influenced by gene architecture and structural properties of promoters
Source: PLoS One. 2019 Mar 25;14(3):e0212678. doi: 10.1371/journal.pone.0212678 (PMC6433290; doi:10.1371/journal.pone.0212678)

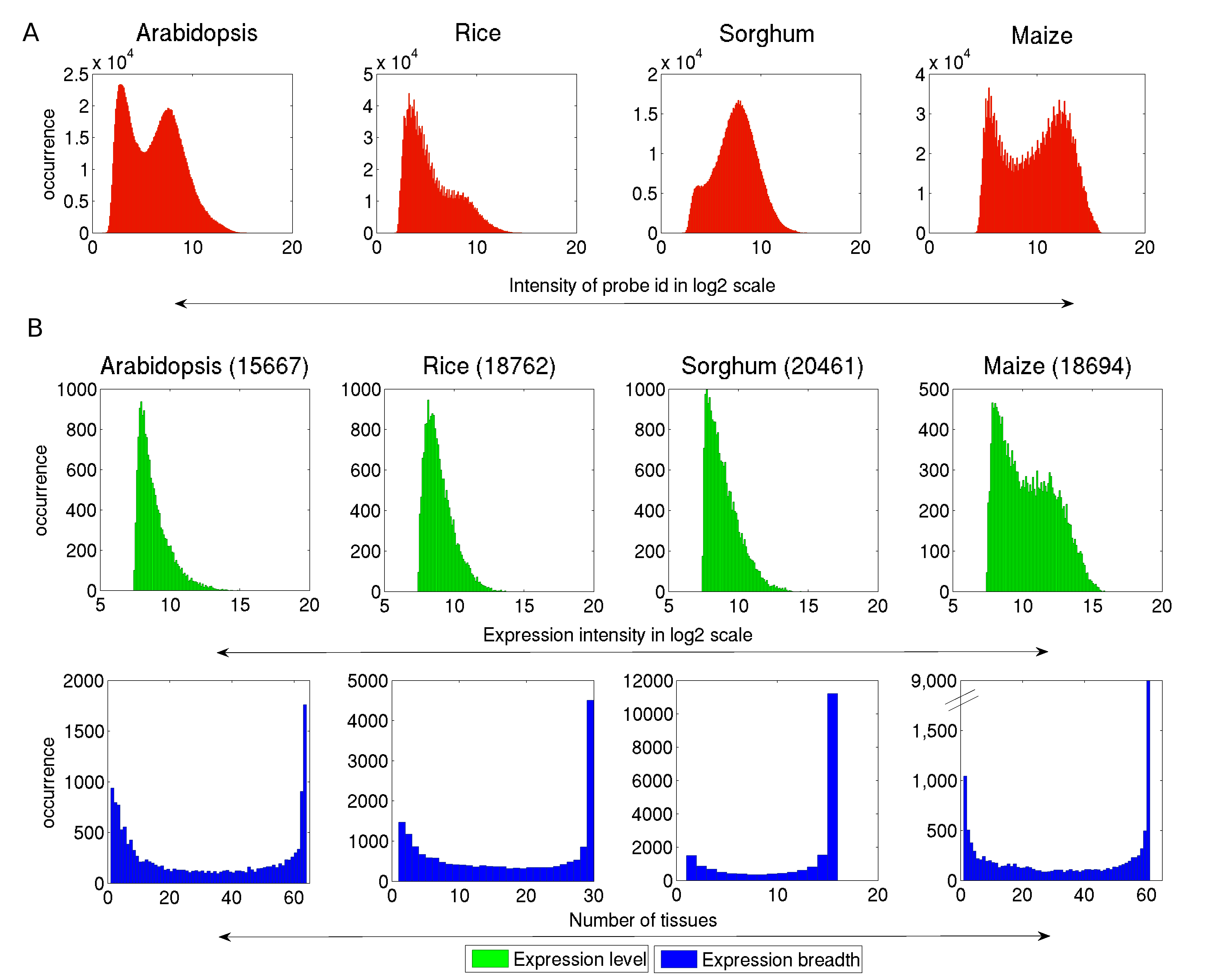

Supplement: S1 Fig — Expression intensity of probe represented on log2 scale (A). Only genes with expression intensity >200 or >7.64 are included for further analysis. (B) The distribution of gene datasets in four plants. A gene is considered as expressed if the expression intensity value is >7.64 (on log2 scale). Histograms are plotted with bin size 1 for both expression level and expression breadth presented in green and blue color respectively. (TIF) [file pone.0212678.s001.tif]

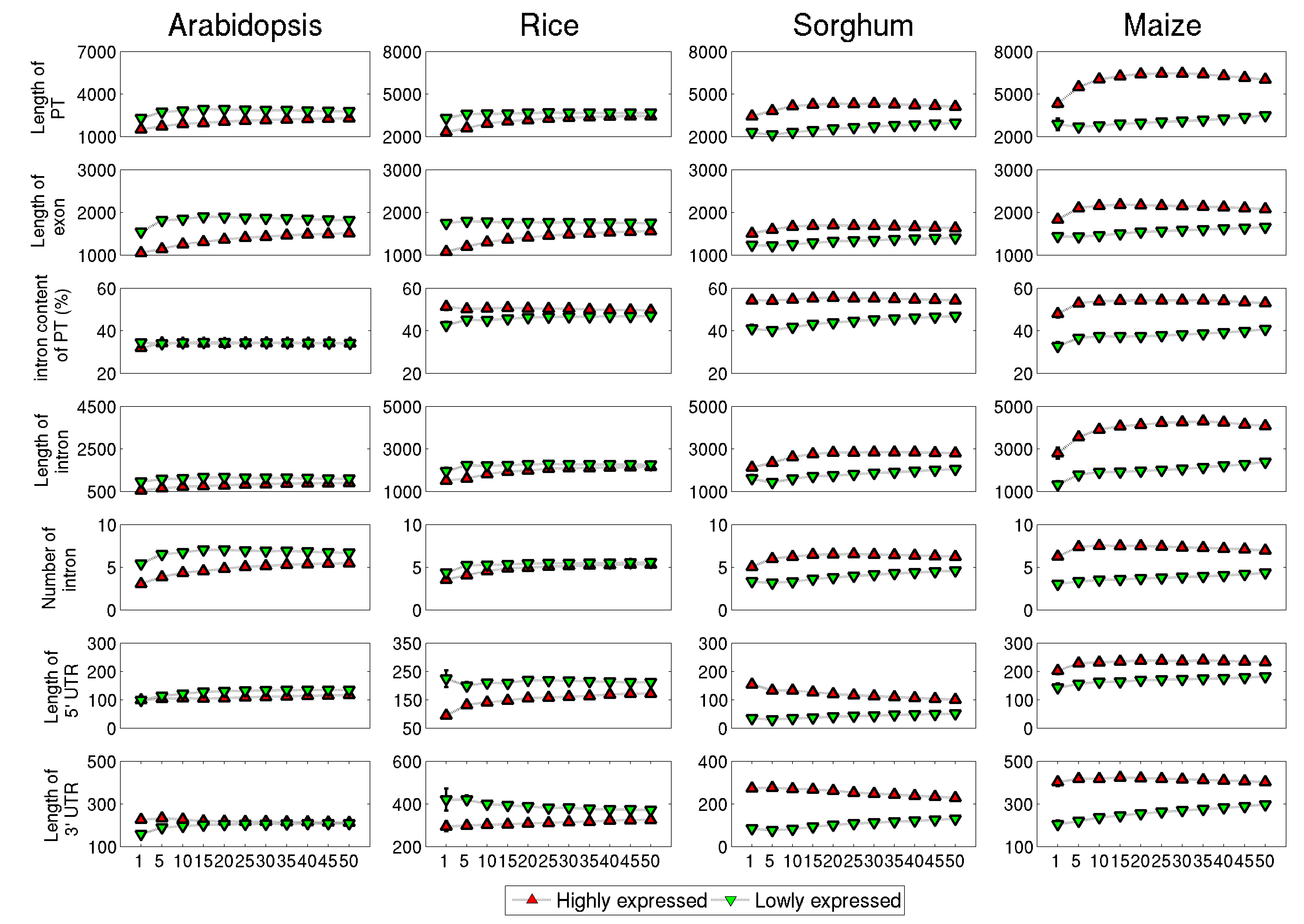

Supplement: S2 Fig — The panel shows the mean of respective parameter values versus the average expression value in 1%, 5%, 10%, 15%, 20%, 25%, 30%, 35%, 40%, 45% and 50% quantiles for both sides of the whole data set in arabidopsis, rice, sorghum and maize. Intron-less genes are removed from this analysis. (TIF) [file pone.0212678.s002.tif]

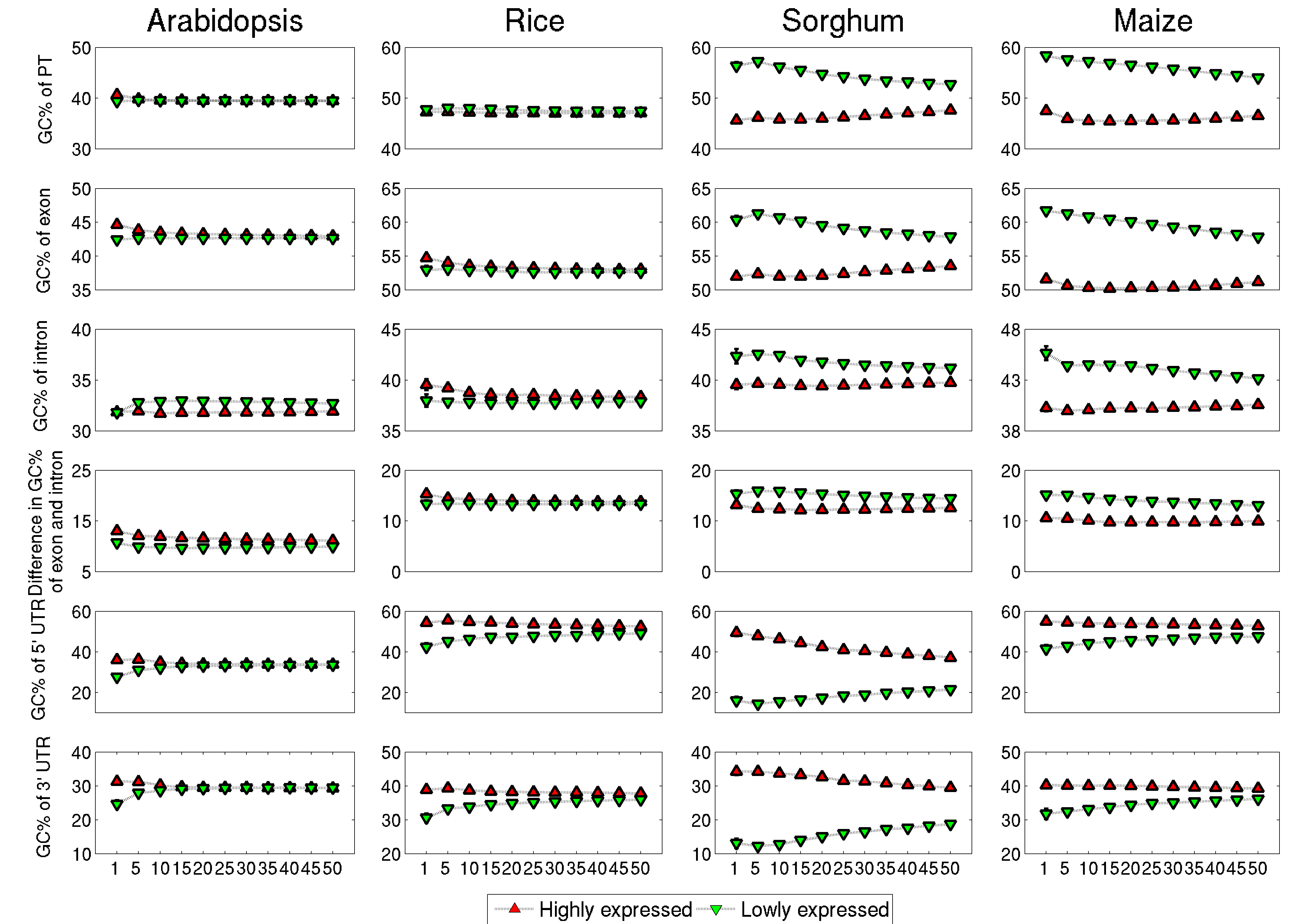

Supplement: S3 Fig — The panel shows the mean of respective parameter values versus the average expression value in 1%, 5%, 10%, 15%, 20%, 25%, 30%, 35%, 40%, 45% and 50% quantiles for both sides of the whole data set in arabidopsis, rice, sorghum and maize. Intron-less genes are removed from this analysis. (TIF) [file pone.0212678.s003.tif]

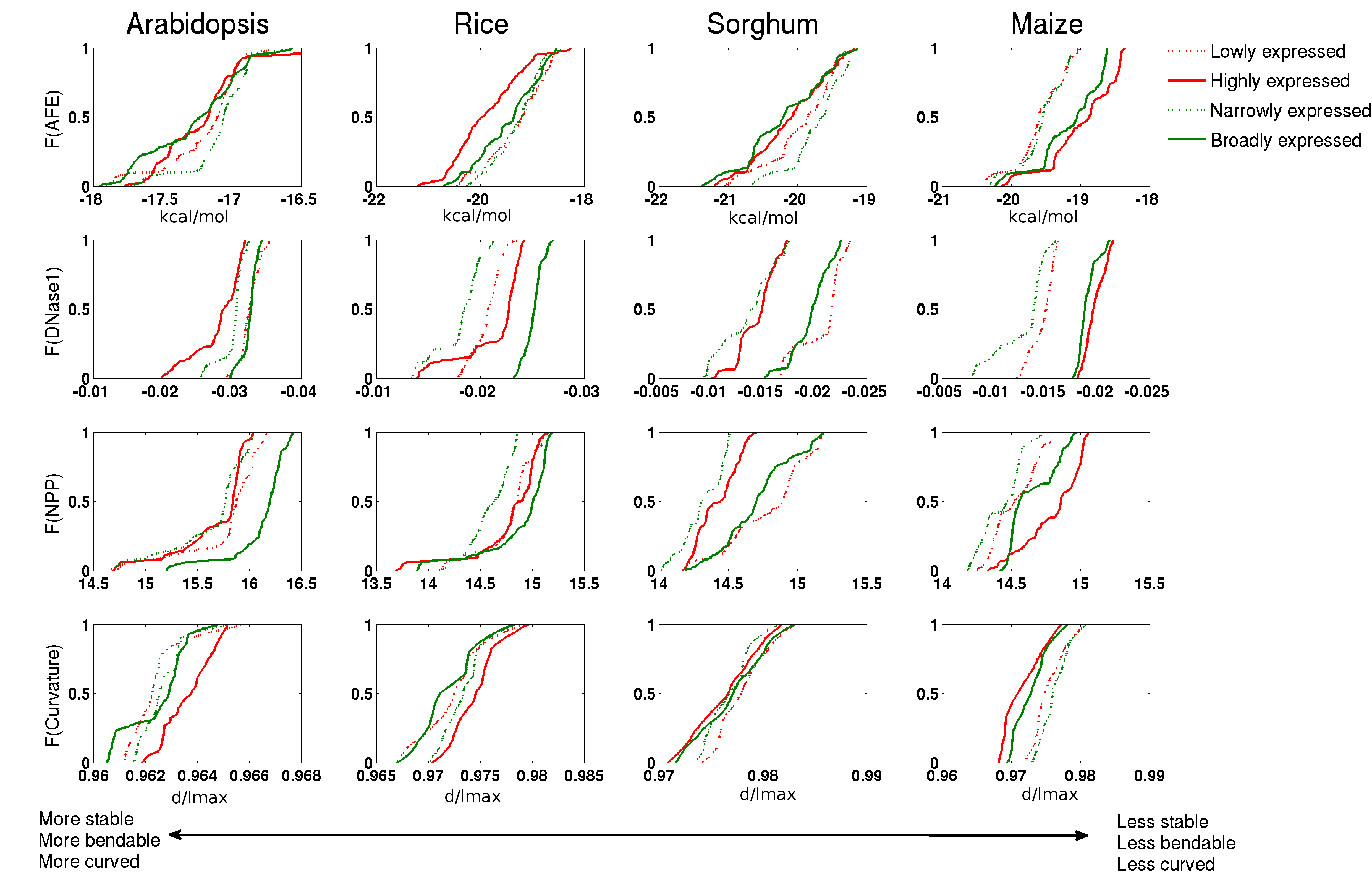

Supplement: S4 Fig — All four properties are presented for arabidopsis, rice, sorghum and maize. The y-axis represents cumulative frequency (value ranges from 0 to 1) and x-axis denotes the values of structural properties. (TIF) [file pone.0212678.s004.tif]

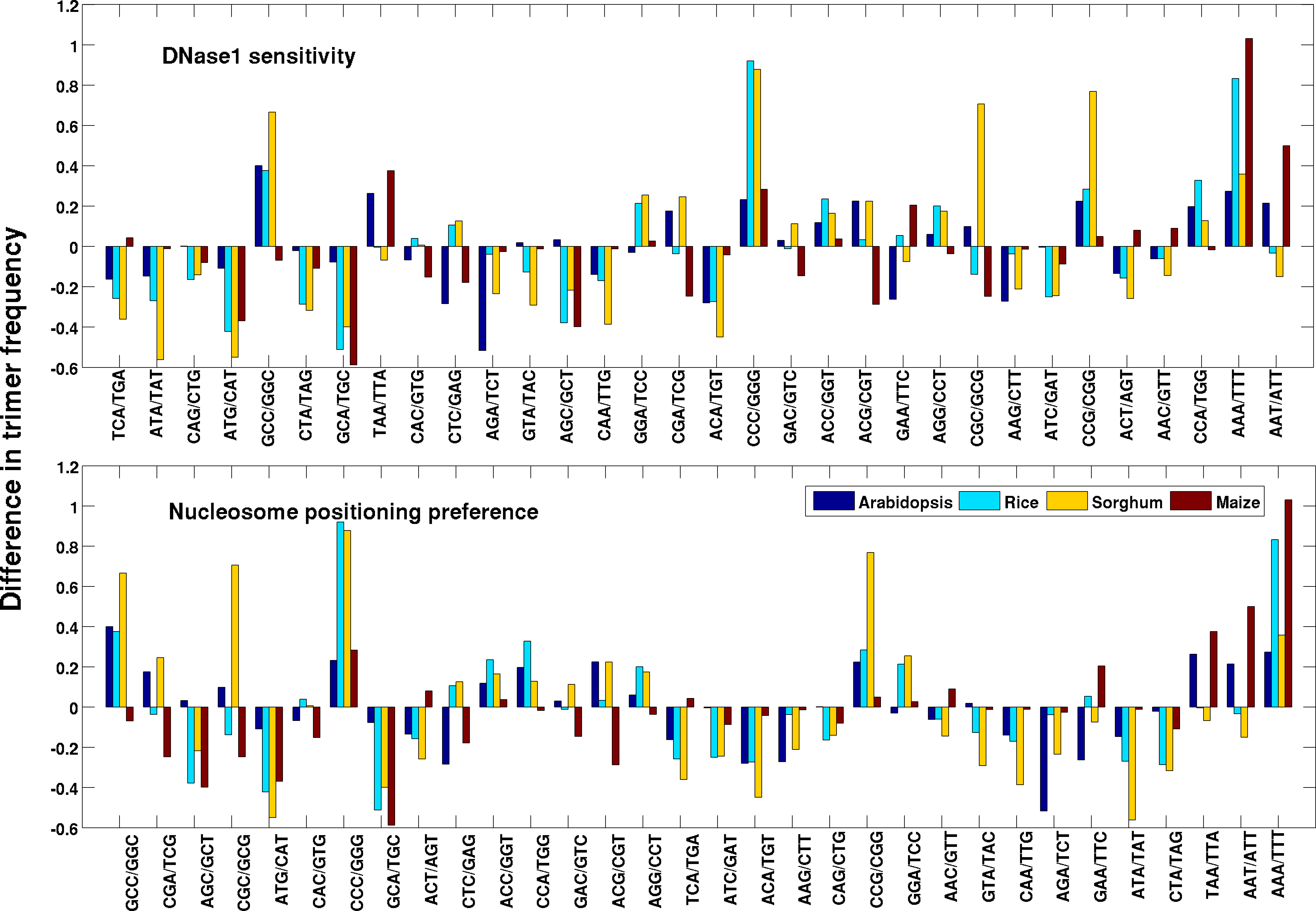

Supplement: S5 Fig — Trimers of four plants are presented in four distinct colors. The top figure shows trimers sorted in order to their flexibility, from high (left) to low (right) for DNase 1 senitivity and the bottom one is for Nucleosome positioning preference sorted in accordance from the minor groove (left) to the major groove (right) with those showing no preference in the middle. Bar present on the positive side correspond to high trimer occurrence in broadly expressed genes. (TIF) [file pone.0212678.s005.tif]

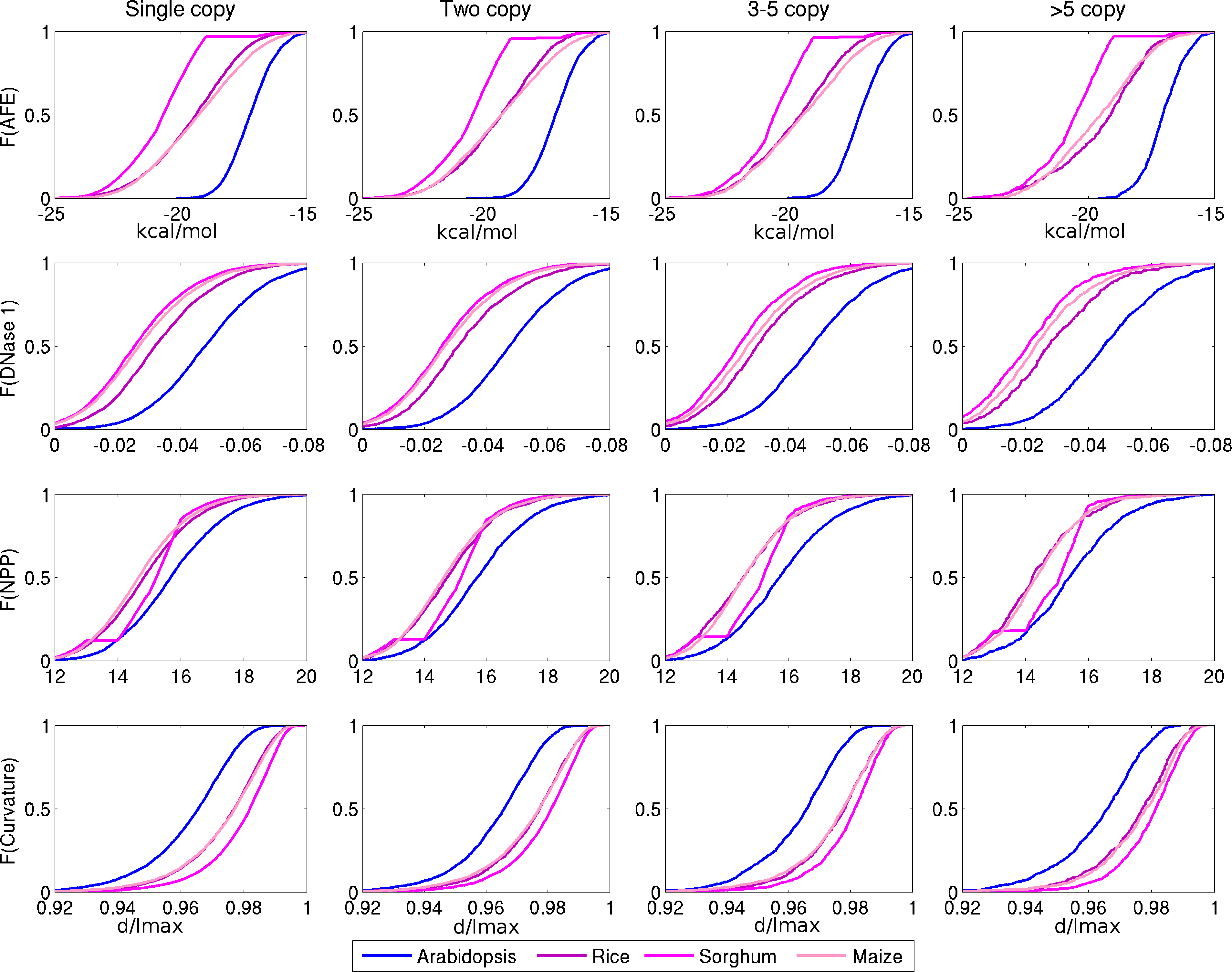

Supplement: S6 Fig — All four properties are presented with an increase in the gene copy number of arabidopsis, rice, sorghum and maize. Similar copy number genes are grouped together and structural properties are calculated for specific plant, re-spective CDFs are juxtaposed for four different plants. The y-axis represents cumulative frequency (values ranges from 0 to 1) and x-axis denotes the values of various structural properties examined. (TIF) [file pone.0212678.s006.tif]

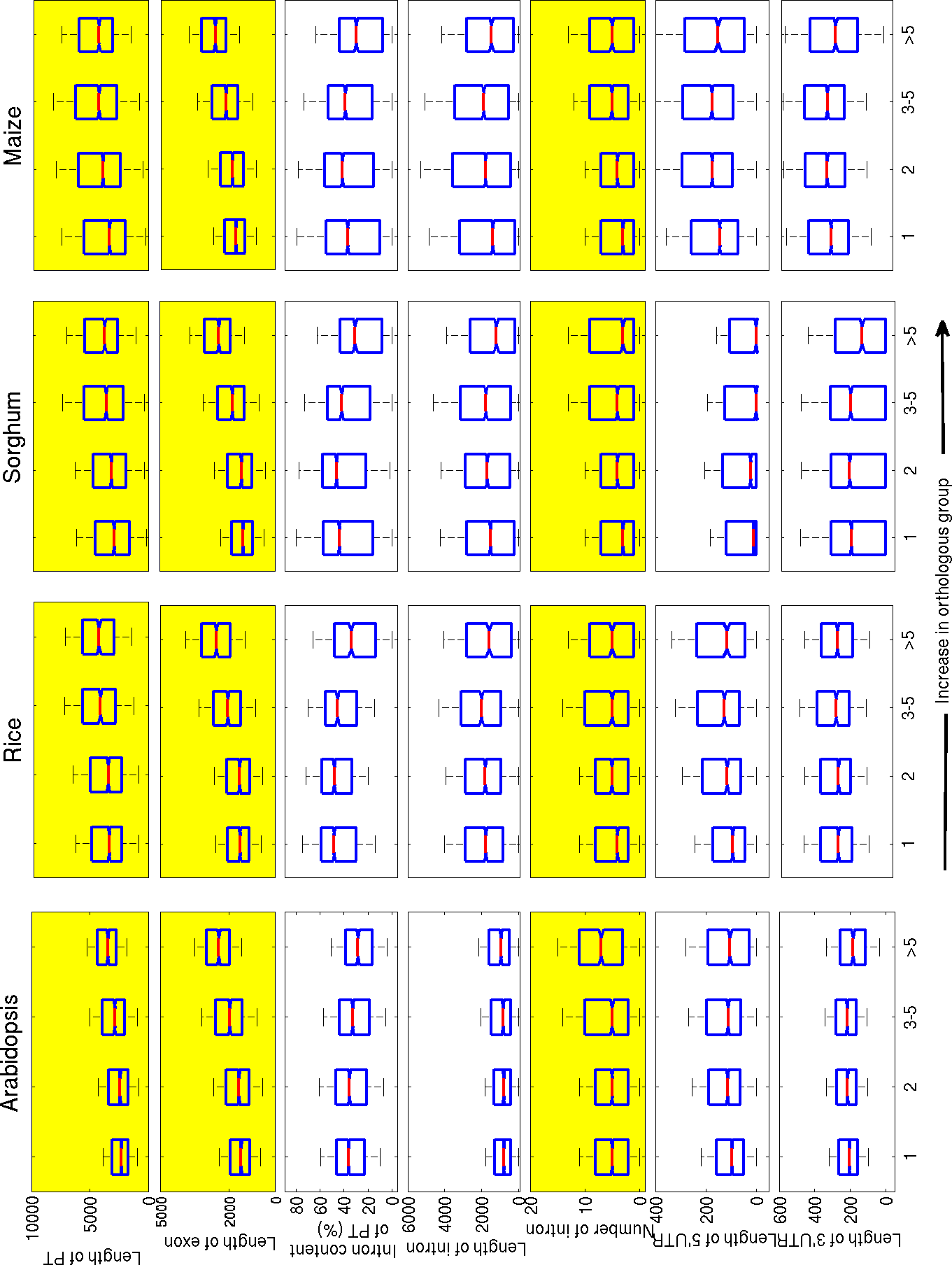

Supplement: S7 Fig — Top and bottom spaces of the box are 25th and 75th percentiles of the dataset with the mid line of the box represent the median position. Outliers are not shown here; nevertheless, these are included during the plotting of box plot. The significant positive relationship between length parameters and orthologous groups are shaded in yellow color. (TIF) [file pone.0212678.s007.tif]

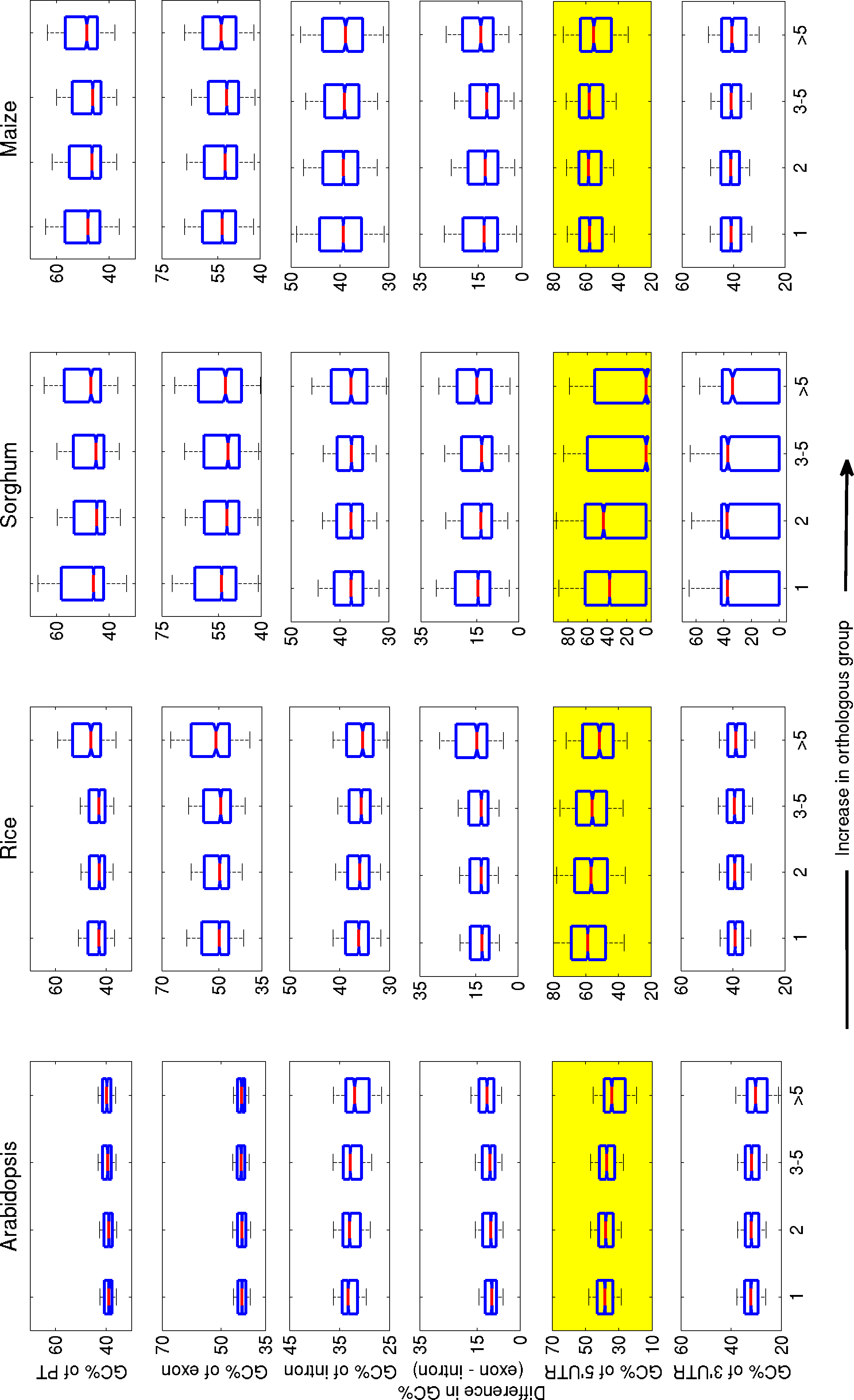

Supplement: S8 Fig — Top and bottom spaces of the box are 25th and 75th percentiles of the dataset with the mid line of the box represents the median position. Outliers are not shown here; nevertheless, these are included during the plotting of box plot. The significant negative relationship between length parameter and orthologous group is shaded in yellow color. (TIF) [file pone.0212678.s008.tif]
