# Supplementary material for: Variation of gene expression in plants is influenced by gene architecture and structural properties of promoters
Source: PLoS One. 2019 Mar 25;14(3):e0212678. doi: 10.1371/journal.pone.0212678 (PMC6433290; doi:10.1371/journal.pone.0212678)
Supplement: S1 Table — After removal of highly correlated gene components with other gene components such as length of intron, length of exon, GC% of PT and difference in GC% of exon and intron. GC% of 5’ UTR and 3’ UTR are removed from Sorghum to reduce the value of VIF of both the respective length of UTRs from 9.6 to 1.7 and 22.0 to 1.9 respectively. (PDF) [file pone.0212678.s009.pdf]

S1 Table. VIF values of parameters after removal of highly correlated gene components.

	Arabidopsis		Rice		Sorghum		Maize	
	Before	After	Before	After	Before	After	Before	After
Length of PT	3.5	2.3	3.2	2.3	3.2	2.5	3.1	2.5
Length of exon	5.9	-	9.8	-	9.6	-	9.9	-
Intron cont.	5.9	1.3	6.1	1.6	6.5	1.8	6.1	2
Intron length	34.3	-	21.2	-	26.3	-	28.4	-
Number of Intron	2.6	2.6	2.2	2	2.1	1.9	2.1	1.7
5’UTR length	2.7	2.6	1.9	1.8	9.6	1.7	3.1	3
3’UTR length	5	5	-	4.1	22	1.9	3.5	3.4
PT GC%	5.9	-	7.1	-	16.6	-	9.1	-
Exon GC%	3004.6	1.1	26639.9	1.9	38711.6	1.9	26236.8	1.9
Intron GC%	63472.8	1.2	123968.1	1.4	125745.3	1.4	139571.7	1.3
Diff. in GC%	64586.6	-	168927.7	-	197887.5	-	187503.6	-
5’UTR GC%	2.8	2.6	1.9	1.8	9.7	-	3.1	3
3’UTR GC%	4.8	4.7	3.8	3.8	20.3	-	3.2	3
AFE	1.9	1.7	1.5	1.4	1.7	1.4	1.8	1.6
DNase 1	1.9	1.8	1.6	1.6	1.6	1.5	1.8	1.7
NPP	2.6	2.5	1.6	1.5	1.3	1.2	1.5	1.4
Curvature	1.2	1.1	1.5	1.5	1.5	1.5	1.6	1.6

After removal of highly correlated gene components with other gene components such as length of intron, length of exon, GC% of PT and difference in GC% of exon and intron. GC% of 5’ UTR and 3’ UTR are removed from Sorghum to reduce the value of VIF of both the respective length of UTRs from 9.6 to 1.7 and 22.0 to 1.9 respectively.
