# Supplementary material for: Variation of gene expression in plants is influenced by gene architecture and structural properties of promoters
Source: PLoS One. 2019 Mar 25;14(3):e0212678. doi: 10.1371/journal.pone.0212678 (PMC6433290; doi:10.1371/journal.pone.0212678)
Supplement: S2 Table — Rice was used as the reference genome to search the single copy, two copies, three to five copies and more than five genes in the queried genome. (PDF) [file pone.0212678.s010.pdf]

S2 Table. Gene families were grouped based on their orthologous copy in the genome.

Orthologous group	Arabidopsis	Rice	Sorghum	Maize
Single copy	4828	3334	9031	6242
Two copy	3111	1548	3480	4495
Five copy	1627	945	1638	2384
More than five copy	692	458	716	951

Rice was used as the reference genome to search the single copy, two copy, three to five copy and more than five genes in the queried genome.
