# Supplementary material for: Variation of gene expression in plants is influenced by gene architecture and structural properties of promoters
Source: PLoS One. 2019 Mar 25;14(3):e0212678. doi: 10.1371/journal.pone.0212678 (PMC6433290; doi:10.1371/journal.pone.0212678)
Supplement: S3 Table — Mean values of parameters are represented with the ± standard deviation of both sides of the data set in arabidopsis, rice, sorghum and maize. Zero intron genes are excluded from the analysis of parameters such as, Intron content of PT (%), length of intron, number of intron, GC% of intron and difference in GC% of intron and exon. (PDF) [file pone.0212678.s011.pdf]

S3 Table. Statistics of parameters of 25% of lowly and highly expressed genes.

	Arabidopsis		Rice		Sorghum		Maize	
Parameters	Lowly expressed	Highly expressed	Lowly expressed	Highly expressed	Lowly expressed	Highly expressed	Lowly expressed	Highly expressed
Length of PT	2874.4±30.3	2123.8±20.6	3684.7±41.7	3278.1±41.4	2641.1±37.2	4312.5±50.5	3032.9±61.6	6461.2±89.5
Lenght of exon	1869.7±17.4	1406.2±13.1	1762.4±13.3	1455.8±11.4	1338.9±10.7	1687.7±14.9	1561.7±10.7	2155.6±15.6
Intron content of PT (%)	34.3±0.2	34.2±0.3	46.5±0.3	50.4±0.3	44.5±0.4	55.3±0.3	37.7±0.4	54.3±0.3
Length of intron	1142.1±17.9	823.7±11.3	2289.9±38.2	2069.7±31.2	1812.7±42.0	2825.7±47.4	2004.8±71.1	4231.5±86.2
Number of intron	6.9±0.1	5.0±0.1	5.5±0.1	5.1±0.1	4.0±0.1	6.5±0.1	3.8±0.1	7.4±0.1
Length of 5'UTR	132.3±2.3	108.9±1.5	218.2±5.9	158.2±3.8	42.5±1.3	115.9±1.8	170.8±3.3	238.2±3.8
Length of 3'UTR	205.3±2.2	216.9±1.9	381.5±7.3	311.9±4.6	107.9±2.3	252.6±2.6	262.6±4.1	418.5±4.5
GC% of PT	39.5±0.1	39.5±0.1	47.5±0.1	47.1±0.1	54.2±0.2	46.3±0.1	56.2±0.1	45.6±0.1
GC% of exon	42.6±0.1	43.2±0.0	52.6±0.1	53.3±0.1	59.1±0.1	52.4±0.1	59.7±0.1	50.3±0.1
GC% of intron	32.9±0.1	31.8±0.1	37.7±0.1	38.6±0.1	41.6±0.1	39.5±0.1	44.2±0.1	40.2±0.1
Difference in GC% of exon and intron	9.7±0.1	11.5±0.1	13.3±0.1	14.0±0.1	15.1±0.1	12.2±0.1	13.9±0.1	9.8±0.1
GC% of 5'UTR	33.3±0.2	33.9±0.2	47.7±0.4	53.6±0.3	18.2±0.4	41.0±0.4	46.0±0.4	53.8±0.3
GC% of 3'UTR	29.3±0.2	29.5±0.1	35.1±0.2	38.1±0.1	15.9±0.3	31.6±0.2	34.9±0.3	39.9±0.2

Mean values of parameters are represented with the ± standard deviation of both sides of the data set in arabidopsis, rice, sorghum and maize. Zero intron genes are excluded from the analysis of parameters such as, Intron content of PT (%), length of intron, number of intron, GC% of intron and difference in GC% of intron and exon.
